# Multitarget Anticancer Agents Based on Histone Deacetylase and Protein Kinase CK2 Inhibitors

**DOI:** 10.3390/molecules25071497

**Published:** 2020-03-25

**Authors:** Regina Martínez, Bruno Di Geronimo, Miryam Pastor, José María Zapico, Claire Coderch, Rostyslav Panchuk, Nadia Skorokhyd, Maciej Maslyk, Ana Ramos, Beatriz de Pascual-Teresa

**Affiliations:** 1 Departamento de Química y Bioquímica, Facultad de Farmacia, Universidad San Pablo-CEU, CEU Universities, Urbanización Montepríncipe, Alcorcón, 28925 Madrid, Spain; reginamartinezflores@gmail.com (R.M.); bru.digeronimo.ce@ceindo.ceu.es (B.D.G.); miryam.pastor.fernandez@univie.ac.at (M.P.); josemaria.zapicorodriguez@ceu.es (J.M.Z.); claire.coderchboue@ceu.es (C.C.); 2 Institute of Cell Biology, NAS of Ukraine, Drahomanov Str. 14/16, 79005 Lviv, Ukraine; rpanchuk@ukr.net (R.P.); n.skorokhyd@gmail.com (N.S.); 3 Department of Molecular Biology, Faculty of Biotechnology and Environment Sciences, The John Paul II Catholic University of Lublin, 20-718 Lublin, Poland; maciekm@kul.pl

**Keywords:** HDAC, CK2, multi-target inhibitors, docking, molecular dynamics, CuAAC, cytotoxic activity

## Abstract

The design of multitarget drugs (MTDs) has become an innovative approach for the search of effective treatments in complex diseases such as cancer. In this work, we communicate our efforts in the design of multi-targeting histone deacetylase (HDAC) and protein kinase CK2 inhibitors as a novel therapeutic strategy against cancer. Using tetrabromobenzotriazole (TBB) and 2-dimethylamino-4,5,6,7-tetrabromo-benzimidazole (DMAT) as scaffolds for CK2 inhibition, and a hydroxamate to coordinate the zinc atom present in the active site of HDAC (zinc binding group, ZBG), new multitarget inhibitors have been designed and synthesized. According to the in vitro assays, *N*-Hydroxy-6-(4,5,6,7-tetrabromo-2-(dimethylamino)-1H-benzo[d]imidazol-1-yl)hexanamide (**11b**) is the most interesting compound, with IC_50_ values of 0.66; 1.46 and 3.67 µM. for HDAC6; HDAC1 and CK2; respectively. Cellular assays on different cancer cell lines rendered promising results for *N*-Hydroxy-8-(4,5,6,7-tetrabromo-2-(dimethylamino)-1H-benzo[d]imidazol-1-yl)octanamide (**11d**). This inhibitor presented the highest cytotoxic activity, proapoptotic capability, and the best mitochondria-targeting and multidrug-circumventing properties, thus being the most promising drug candidate for further in vivo studies.

## 1. Introduction

The development of new efficient anticancer drugs still represents a challenge for medicinal chemists. Although significant progress has been made, single-target drugs still have significant drawbacks, such as induced toxicity and drug-resistance. Combination cancer therapy, a modality that combines two or more agents acting on different pharmacological targets, has expanded the treatment options. These therapies achieve the desirable results in a concerted manner through different mechanisms of action [1,2,3]. Nevertheless, anticancer drug cocktails have also some disadvantages, such as the possibility of drug–drug interactions, pharmacokinetic complexity, and low patient compliance [4,5]. As an alternative, mutitarget drugs (MTDs) have emerged as a novel strategy to overcome all these multicomponent cocktail limitations [6,7]. Moreover, multitarget drug discovery has economic advantages because it requires less clinical trials than multiple specific drugs. For all these reasons, the design of a single molecule, which can efficiently inhibit more than one biological target, constitutes an interesting and promising challenge in modern pharmacotherapy [8,9]. In fact, in the recent past years, several research groups have published successful dual inhibitors for the treatment of different disorders such as cancer, diabetes, psychiatric pathologies, inflammation and neurodegenerative diseases [10,11,12,13].

**Figure 1:**
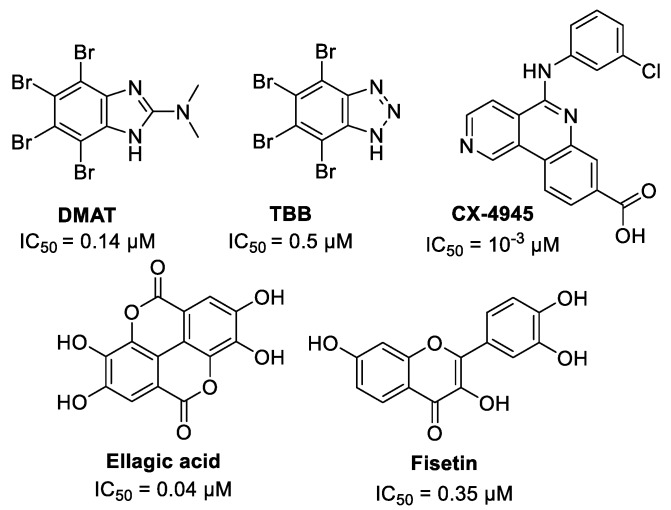
Representative CK2 inhibitors.

Protein kinase CK2 is a pleiotropic and ubiquitous serine/threonine protein kinase, which is composed of tetrameric complexes consisting of two catalytic subunits (CK2α, CK2α’) and a dimer of the regulatory β subunits [14,15]. CK2 phosphorylates a wide variety of substrates [16] and is essential for cell viability. It is localized in different cell compartments and is implicated in several essential functions, including signal transduction, cell growth and differentiation, gene expression and apoptosis [17,18]. CK2 is involved in several human pathologies, among them neurodegeneration and cancer. A wide variety of cancer cells show elevated levels of its enzymatic activity [19], and many cancers, including kidney, head and neck, mammary gland, lung, and prostate show overexpression of CK2 [20]. Anti-apoptotic and proliferative properties of this kinase create a favorable cellular environment for tumor maintenance and progression. For all these reasons, CK2 has emerged as a potential oncology target [21]. To date, several compounds, which potently inhibit CK2, have been described [22,23,24]. Among the ATP-competitive CK2 inhibitors reported, ellagic acid, coumarin derivatives, DMAT, TBB and Silmitasertib (CX-4945) (Figure 1) are distinguished by their high efficiency [25,26,27,28,29,30,31,32,33]. Benzonaphthyridine derivative CX-4945 is one of the most potent CK2 inhibitors and is currently in phase II clinical trials [34,35] (Figure 1).

**Figure 2:**
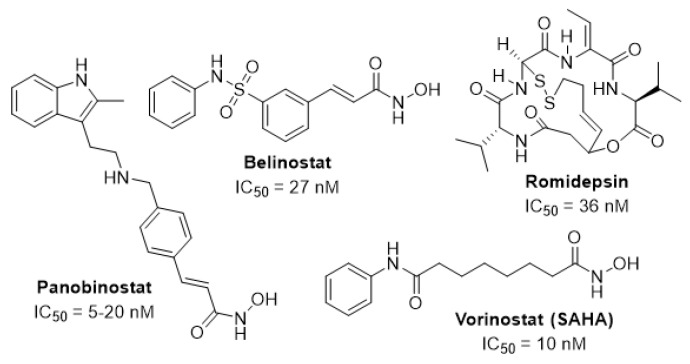
FDA-approved histone deacetylase (HDAC) inhibitors [50].

Histone deacetylases (HDAC) are a type of epigenetic enzymes that in recent years have aroused great interest in oncology. Its aberrant activity has been related to cell proliferation in a variety of human pathologies, especially solid tumors and myeloid neoplasia [36,37,38,39,40,41]. In humans, there are 18 HDAC isoforms that are divided into four different classes (I–IV). Class I includes HDAC1, 2, 3 and 8; class II includes 4, 5, 6, 7, 9 and 10; class III is made up of members of the sirtuin family and class IV is represented by HDAC11. Except class III, they are Zn-dependent enzymes. Class I HDACs are ubiquitously expressed and play a critical role in proliferation, while classes II and IV have tissue-specific roles [42]. These enzymes catalyze the deacetylation of the amino group of lysine residues in the N-terminal tails of histone and non-histone proteins and are involved in cell cycle progression, transcriptional regulation and apoptosis [43,44]. For a normal cell growth, a balance between acetylation by histone acetyl transferases (HATs) and deacetylation by HDACs is essential. HDAC inhibitors (HDACi), by inducing hyperacetylation of histones, alter gene transcription and exert antitumor effects through differentiation, growth arrest or/and apoptosis [45,46]. HDAC inhibition has become an important strategy in epigenetic drug discovery, and many HDAC inihibitors are currently under clinical investigation for cancer therapy [47,48]. Vorinosat (SAHA), Romidepsin (FK-228), Belinostat (PXD101) and Panobinostat (LBH589) have been approved by the FDA for the treatment of specific lymphoma and myeloma diseases [49] (Figure 2). 

Some examples of multitarget inhibitors that combine structural features of HDACi and other anticancer agents have been published in the recent years [51,52,53,54,55,56,57,58]. Combination of kinase inhibitors with the HDAC pharmacophore has been recently reported [59,60]. Two hybrid compounds are currently in clinical trials: an Erlotinib-based hybrid with activity in HDACs, EGFR and HER2 (IC_50_ = 4.4, 2.4 and 15.7 nM, respectively), and Fimepinostat (CUDC-907), which is an orally active pan PI3K/HDAC inhibitor [61].

Protein kinase CK2 constitutes an attractive option for a secondary target, owing not only to its role in the regulation of HDACs by post-translational modification [62,63], but also to its capacity to activate HDAC1 in tumors associated with hypoxia [64,65].

**Scheme 1:**
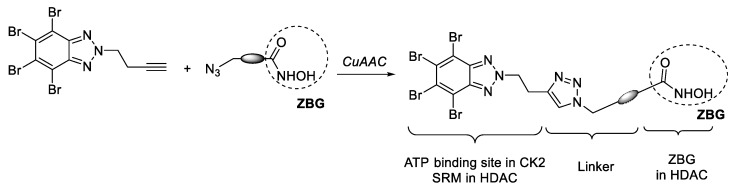
General approach for the synthesis of dual HDAC/CK2 based on TBB.

In this work, we communicate our efforts to develop new dual HDAC/CK2 inhibitors with utility as anticancer agents. With this aim, we selected TBB and DMAT (Figure 1), which are ATP-competitive inhibitors with a high affinity for the ATP binding site of CK2. These structures were functionalized with a terminal alkyne moiety and were connected to another subunit designed to interact with HDAC. This subunit is provided with an hydroxamic acid to act as a zinc-binding group (ZBG), and an azide for the Cu(I)-catalyzed alkyne azide cycloaddition reaction (CuAAC) (Scheme 1). On the other hand, the fragment of TBB or DMAT, besides occupying the ATP binding site in CK2, can interact with the residues in the rim of the active site in HDAC1, acting as a surface recognition moiety (SRM).

**Figure 3:**
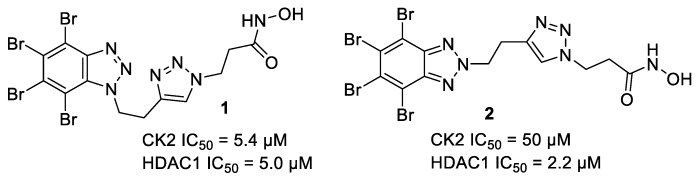
Examples of previously described CK2/HDAC1 dual inhibitors [30].

Previous results in our research group show that this strategy is valid. A series of compounds was synthesized, and promising inhibitors with an IC_50_ value in the order of 5 µM against both enzymes were reported [30]. Although the best inhibitory activity was obtained for compound **1**, the corresponding N_2_-substituted isomer **2** (Figure 3) presented the best profile in cell-based assays, with cytotoxic activity in the low micromolar LC_50_ in two mammalian cell lines. Furthermore, this hybrid molecule induced apoptosis in leukemia cells in a concentration-dependent manner.

## 2. Results and Discussion

### 2.1. Chemistry

The first modification that we introduced in the previously described inhibitors **1–2** (Figure 3) was the substitution of the TBB core by a 2-dimethylaminobenzoimidazole moiety, which is present in DMAT, a more potent and selective CK2 inhibitor (Figure 1).

**Scheme 2:**
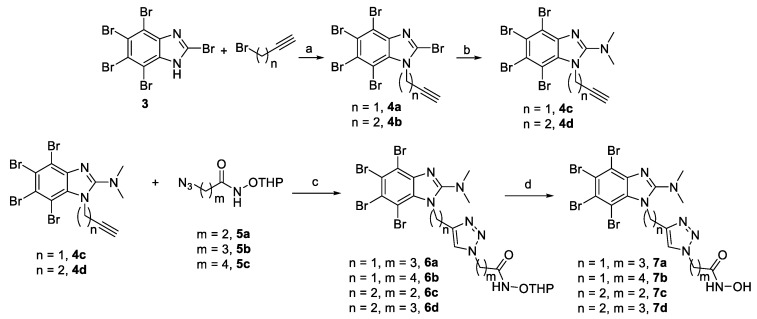
Synthetic route for compounds **7a–d**. Reaction conditions: (**a**) K_2_CO_3_, acetone, MW, 110 °C, 60 min; (**b**) HNMe_2_, MeOH, 110 °C, 48 h; (**c**) CuSO_4_, sodium ascorbate, TBTA, RT, overnight; (**d**) AcCl, MeOH, 0 °C, 15 min.

Connecting imidazole chains of different lengths were used in the design of hydroxamates **7**. A convergent strategy was used for the synthesis of these compounds, involving a CuAAC [66] as the crucial step to connect HDAC and CK2 inhibition moieties (Scheme 2). Alkynes **4a–b** were easily prepared by alkylation of polybrominated benzimidazole **3** [67] with the corresponding alkynyl bromide in the presence of K_2_CO_3_ [68]. Treatment of **4a–b** with dimethylamine, yielded DMAT-derived alkynes **4c–d.** Azides **5** were prepared according to our previous work [30]. CuAAC between azides **5a–e** and the two above mentioned alkynes **4c–d** using tris(benzyltriazolylmethyl)amine (TBTA) as co-catalyst, gave derivatives **6**. Deprotection of the hydroxamate group gave compounds **7** with moderate to good yields and with a purity higher than 95% (HPLC) after column chromatography.

**Scheme 3:**
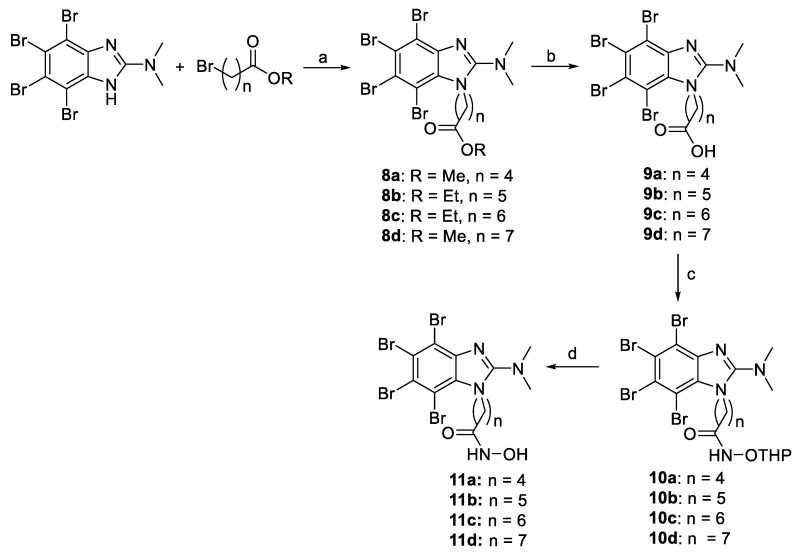
Synthetic route for compounds **11a–d**. Reactions conditions: (**a**) K_2_CO_3_, acetone, reflux, 72 h; (**b**) LiOH, THF/H_2_O, RT, 2h; (**c**) H_2_N-OTHP, EDCI, HOBt, NMM, DMF, RT, overnight; (**d**) AcCl, MeOH, 0 °C, 15 min.

To analyze if the presence of the triazole ring is mandatory for the inhibitory activity in one or both enzymes, DMAT-derivatives **11**, containing hydrocarbon chain linkers of different sizes were synthesized. As depicted in Scheme 3, *N*_1_-alkylation of DMAT by reaction with the corresponding bromoester, followed by hydrolysis gave acids **9**, which were coupled with THP *O*-protected hydroxylamine to give **10**. Finally, deprotection of the hydroxamate group using in situ generated hydrochloric acid gave compounds **11**.

**Figure 4:**
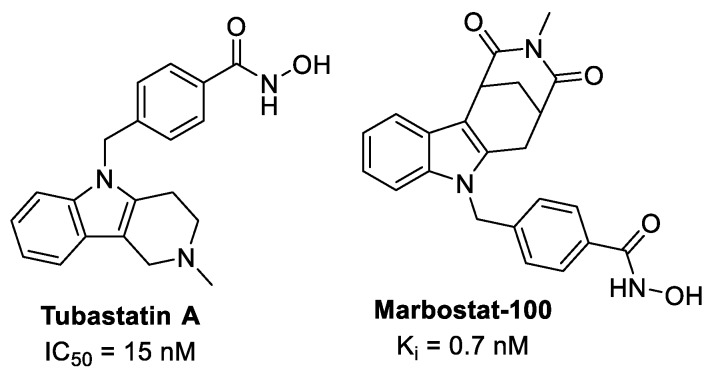
Phenylhydroxamate-based HDAC-6 inhibitors.

Many phenylhydroxamate-based HDAC inhibitors have been described, showing high potency and selectivity, especially for HDAC6 [69]. These structures are exemplified by Tubastatin A [70] and more recently by Marbostat-100 [71], a highly selective and potent inhibitor of HDAC6, being superior to the established HDACi Tubastatin A (Figure 4). HDAC6 has no in vivo activity against histones, but its targets are tubulin, HSP90 and other proteins involved in cancer development and progression [71]. Recently, a JAK2/HDAC6 dual inhibitor was identified to possess potent antiproliferative activity towards hematological cell lines and excellent in vivo antitumor efficacy in several acute myeloid leukemia models [72].

**Scheme 4:**
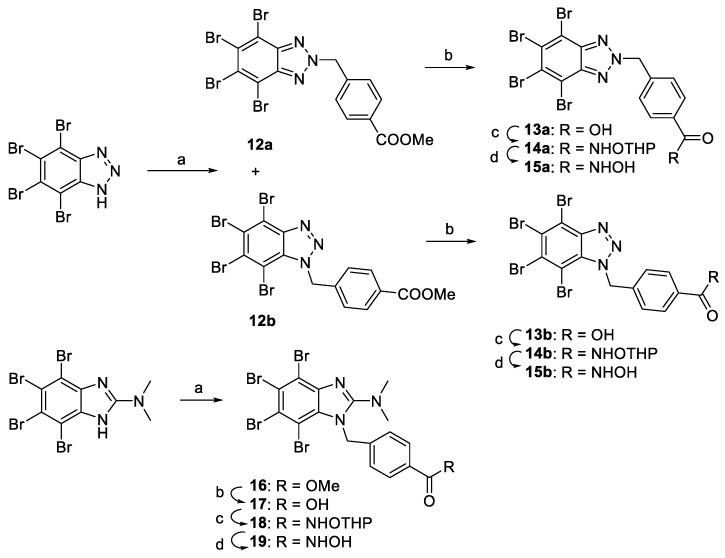
Synthetic route for compounds **13–19**. Reactions conditions: (**a**) methyl 4-(bromomethyl)benzoate, K_2_CO_3_, acetone, 150 °C, MW; (**b**) NaOH, THF, RT; (**c**) H_2_N-OTHP, EDCI, HOBt, NMM, DMF, RT, 24 h; (**d**) AcCl, MeOH, 0 °C, 15 min.

Taking this into account, compounds **15a–b** and **19** derived from TBB [73] and DMAT [74] respectively were synthetized (Scheme 4). *N*_1_-alkylation of the corresponding CK2i scaffold with methyl 4-(bromomethyl)benzoate was followed by hydrolysis, furnishing acids **13** and **17**, which were coupled with THP O-protected hydroxylamine to obtain **14** and **18**. Deprotection of the hydroxamate group gave final products **15a–b** and **19**.

### 2.2. Enzymatic Inhibitory Evaluation

The inhibitory activities of the synthesized compounds were determined against CK2 and HDAC1 and the IC_50_ values are collected in Table 1. The activity towards human recombinant HDAC1 was tested using a fluorometric method. CK2 inhibitory activity was determined by a radiometric method, using a short peptide (RRRADDSDDDDD) as a substrate.

Compounds derived from DMAT were active in both enzymes. Compounds **7a** and **7b**, bearing a triazole ring in the connecting chain, presented an interesting activity in HDAC1 (1.43 and 2.25 µM), but they were 60-fold and 22-fold less active in CK2 respectively. A better result was obtained for compound **7c**, which is a DMAT analogous of the best TBB-derivative previously described by our group [30]. In this case, the activity in both enzymes was very similar, which is the characteristic sought for a dual inhibitor. The introduction of another carbon atom between the imidazole ring and the hydroxamate (compound **7d**) brought about a decrease in CK2 inhibitory activity.

In the series of compounds with alkyl connecting chains, the best result was obtained for **11b**. The activity against HDAC1 (IC_50_ = 1.46 µM) and CK2 (IC_50_ = 3.67 µM) has improved our previous results on TBB derivatives **1** and **2**. Longer chains of six and seven carbon atoms (**11c** and **11d**) were also active, although **11c** presented a 4.5-fold difference in the CK2 inhibitory activity compared to **11b**, and **11d** presented a ninefold difference with **11b** in the HDAC1 inhibitory activity. Compound **11a,** provided with a four-carbon atom chain, was less active in both enzymes. These results demonstrate that the presence of the triazole ring is not essential for inhibitory activity in this series of DMAT derivatives.

The introduction of an aromatic ring in the connecting chain of TBB derivative **15a** and DMAT derivative **19** gave also interesting dual HDAC1/CK2 inhibitors, with IC_50_ in the low micromolar range. We had foreseen that these compounds, carrying an aryl hydroxamate such as the one present in Tubastatin A and Marbostat 100 (Figure 4), could be active in HDAC6. For this reason, we analyzed the activity of **15** and **19**, together with other compounds from the alkyl and triazole series in HDAC6, tested using a fluorometric method (Table 1). Interestingly, we found that all of them were active in HDAC6, with the best result for **11b** with IC_50_ of 0.66 µM. This compound presented also an interesting activity in HDAC1 and CK2 with IC_50_ values of 1.46 and 3.67 µM, respectively.

In summary, we have found interesting HDAC/CK2 multitarget compounds, which can inhibit three enzymes involved in proliferative events through different mechanisms. 

### 2.3. Molecular Modelling

Computational modeling techniques were applied in order to rationalize the biological results shown in Table 1. For this purpose, docking techniques were used as a first approach, followed by Molecular Dynamics (MD) simulations with the aim of proposing a plausible binding mode for the complexes on the three enzymes, CK2, HDAC1 and HDAC6, and of evaluating the stability of the ligand-target complexes in an aqueous and dynamic environment.

**CK2.** The general binding mode of known selective inhibitors such as TBB, TBI and DMAT in CK2 has been established through X-ray studies [75,76,77]. Upon binding, these ligands occupy the adenine portion of the ATP through strong and selective interactions. Docking calculations performed in PDB code 5CQU as protein target with our set of compounds predicted an overall similar binding mode for the TBB and TBI moieties, that slightly differs from the reference TBB moiety of the bound ligand (**JRJ**) in the 5CQU structure [29]. The main differences arise from the orientation of the linker and the hydroxamic acid moiety. The general predicted binding mode of compounds **11a–d, 7a, 7c, 7d, 15a–b** and **19**, has the hydroxamic acid pointing towards the catalytic loop (Figure S1, SI), establishing different hydrogen bonds with the side chains of Lys158, Asn161, and Asp175. The minor differences among the predicted binding modes of these compounds come from the length and adjustment of the aliphatic or aromatic linkers inside the ATP binding site. Compounds **15a–b**, (Figure S2, SI) could reach the catalytic loop through a slight rotation of the tetrabromo benzyl moiety, differently from compounds **11a–d, 7a, 7d** and **19,** that preserved the position of all bromine atoms when compared to **JRJ** from the reference crystal structure. Unlike the latter compounds, compound **7b** is predicted to bind CK2 with the hydroxamic acid pointing towards the αD pocket and establishing hydrogen bond interactions with the side chains of Asn118 and Asp120, and the backbone of His160 (Figure S1, SI).

To better understand the stability of the different predicted binding modes and rationalize the in vitro results, all complexes were submitted to 20 ns Molecular Dynamics (MD) simulations and the ligand RMSD values were monitored along the simulation time (See Table S1, SI). The predicted binding mode of compounds **7a** and **7d** and **11a–d** is preserved along the MD simulation, giving a mean RMSD value lower than 2.5 Å with only slight variations between them. These compounds establish a stable hydrogen bond between the hydroxamic acid and the side chain of Asp156 from the catalytic loop; whereas compound **7c** was not able to maintain this hydrogen bond (Figure S3, SI). Compound **7b** maintained the interaction with the αD pocket stabilized by the transient hydrogen bond with the side chain of Asn118, and a more stable hydrogen bond with the backbone of Thr119. (Figure S4, SI). In line with their smaller size, compounds **15a, 15b** and **19** presented the lowest RMSD values (under 1.6 Å). However, only the latter located the four bromine atoms of the DMAT moiety in the same orientation as the reference structure **JRJ**, whereas compounds **15a** and **15b** needed to, either twist the TBB moiety, or relocate the bromine atoms in a different way (Figure S2, SI).

HDAC1 and HDAC6. We studied the general predicted binding mode of our set of compounds by docking techniques, and assessed the stability of their predicted binding mode by means of MD simulations. The overall behavior of these compounds in complex to HDAC1 and HDAC6 did not differ from those found in the literature [30]. All compounds established a bidentate chelation of the catalytic Zn^2+^, which was ion-, stabilized by hydrogen bonds with the side chains of His140/His610 and Tyr303/Tyr782 in HDAC1 and HDAC6, respectively (Figure S5, SI). The aliphatic linker interacted with the side chains of Phe205/Phe620 and Phe150/Phe680, both of which line the access tunnel to the metal site of both enzymes, and oriented the SRM to interact with amino acids in the surface of the protein. Thus, in the complexes with HDAC1, the aliphatic chain of compounds **11a–b** and **11d** oriented the DMAT moiety towards the side chains of Tyr204 and Phe205, allowing the establishment of π-π interactions; whereas for compound **11c** the DMAT moiety was oriented to interact with the side chains of Leu271 and Phe150 (Figure S6, SI). The complexes of **11a–d** with HDAC6 presented an overall similar predicted binding mode, which can be divided into two subgroups: compounds **11a** and **11d** interact with Phe679 and Met682, whereas compounds **11b** and **11c** interact with His500, Pro501 and Leu749 (Figure S7, SI). Compounds **7a–d**, bearing the triazol linker, established a sandwiched π-π interaction with the side chains of Phe205/Phe620 and Phe150/Phe680, in HDAC 1 and 6, respectively. Their SRMs interact indistinctively with the amino acids of the perimeter of the entrance of the tunnel (Figures S6 and S7, SI). Despite their smaller and shorter structures, compounds **15a–b** and **19** were able to reach the metal site, establishing strong π-π interactions with the side chains of Phe205/Phe620 and Phe150/Phe680, in HDAC 1 and 6, respectively. However, in this case, the TBB and DMAT moieties interacted, either with Phe205 and Leu271 on the surface of HDAC1, or with Pro501, Phe679, Met682 and Leu749 in HDAC6, all of them close to the tunnel entrance (Figures S6 and S7, SI).

During the MD simulations of the HDAC1 and HDAC6 complexes, the general behavior of the bound compounds was the exploration of the surface of the protein by the SRM, while the hydroxamate and the linker remained stably bound to the catalytic site of the HDACs. The largest RMSD values corresponded to compounds **7a–d**, mainly due to the flexibility of the linker that exposed the SRM to the surface of the protein. This was translated into transient interactions with amino acids in the surface of the protein but with no clear destabilization of the predicted binding mode. Compounds **11a–d** showed small rearrangements from the starting orientations but preserved the initial interactions described above along the simulation time. Compounds **15a–b** and **19** presented small RMSD variations, in line with their small size, which lead to similar behavior to that of the CK2 complexes (Tables S2 and S3, SI).

**Figure 5:**
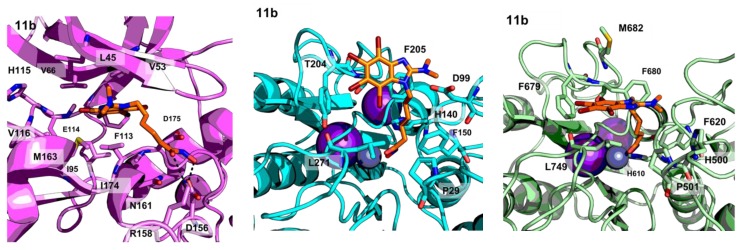
PyMOL stick and cartoon representation of the proposed binding modes of compound **11b** to CK2 (magenta), HDAC1 (cyan) and HDAC6 (light green). For the sake of clarity, only heavy atoms are shown.

The in vitro activity of compound **11d** is in agreement with the obtained models (Figure 5).

The interaction of the SRM with CK2 does not vary from that in the reference crystal structure (PDB code 1ZOE), despite the presence of the linker and the hydroxamate group. The latter establishes additional hydrogen bonding interactions with the protein target. As for the binding to the HDACs, the hydroxamate establishes a bidentate chelation with the catalytic zinc ion such as the one found for SAHA (PDB code 1T69), while the linker allows the SRM to interact with the surface of the proteins in a way similar to that reported in the literature [30].

Additionally, the pan-assay interference compounds properties (PAINS) were examined by using the SWISS-ADME web server [78]. Interestingly, all compounds showed neither potential PAINS nor other promiscuity alerts, which makes them good drug candidates.

The best dual enzymatic inhibitors (**11b–d**), a representative of the triazole series (**7c**) and two aromatic linker containing compounds (**15a** and **19**), were selected for in vitro studies.

### 2.4. In Vitro Evaluation

#### 2.4.1. Proapoptotic Activity Against Tumor and Pseudonormal Cell Lines.

**Figure 6:**
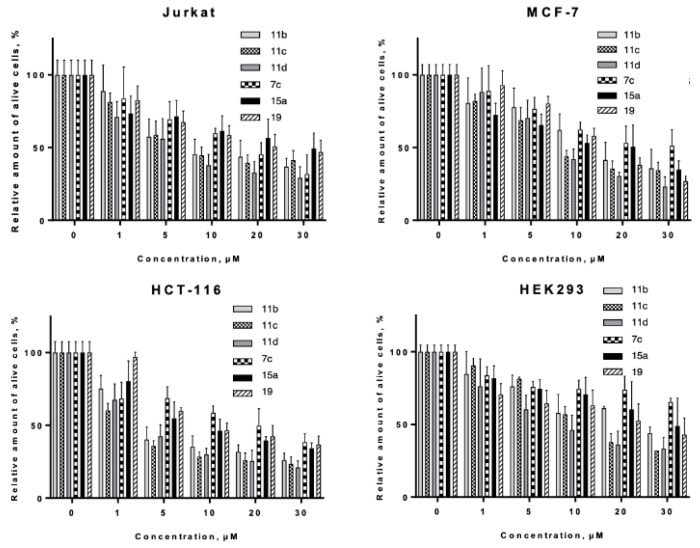
Comparison of cytotoxic activity of selected CK2-HDAC dual inhibitors (**7c**, **11b, 11c, 11d, 15a** and **19**) towards human leukemia (top left), carcinoma (top right and down left,) and pseudonormal (down right) cell lines. Trypan blue assay, 24 h incubation. Means and SD of at least three experiments in triplicate are shown.

Cytotoxic activity of selected CK2-HDAC multitarget inhibitors was investigated in vitro towards human leukemia (Jurkat) and carcinoma (MCF-7, HCT-116) cell lines using trypan blue exclusion assay. For the evaluation of potential cancer selectivity of the selected compounds, their activity was also tested towards human embryonic kidney cells of HEK293 line under identical conditions (Figure 6). LC_50_ doses (concentration of drug killing 50% cells) of all compounds are summarized in Table 2.

DMAT-derived hydroxamates **11b–d** showed twofold higher activity towards various types of tumor cells compared to derivatives containing aromatic linkers (**15a** and **19**) (Figure 6, Table 2). Moreover, the length of the aliphatic chain in **11b–d** inhibitors influenced their cytotoxic activity towards tumor cells. In particular, **11b** with a five carbon aliphatic chain was the weakest compound (LC_50_ = 4.22–23.33 µM depending on cell line), while **11c** (six-carbon chain) demonstrated higher activity (LC_50_ in 1.90–13.61 µM range), and **11d** (seven-carbon chain) was the most active among all tested dual inhibitors (LC_50_ in 2.32–9.02 µM range). However, introduction of a triazole ring in the linker of DMAT derived hydroxamates (**7c**) significantly decreased the antiproliferative activity towards all tested cell lines, lowering it down to the level of CK2-HDAC inhibitors with aromatic linkers (**15a** and **19**). HEK293 cell line was found to be two to three times more resistant to action the action of DMAT derived hydroxamates compared to leukemia and carcinoma cells, thus indicating of targeted action of synthesized inhibitors towards tumor cell lines.

**Figure 7:**
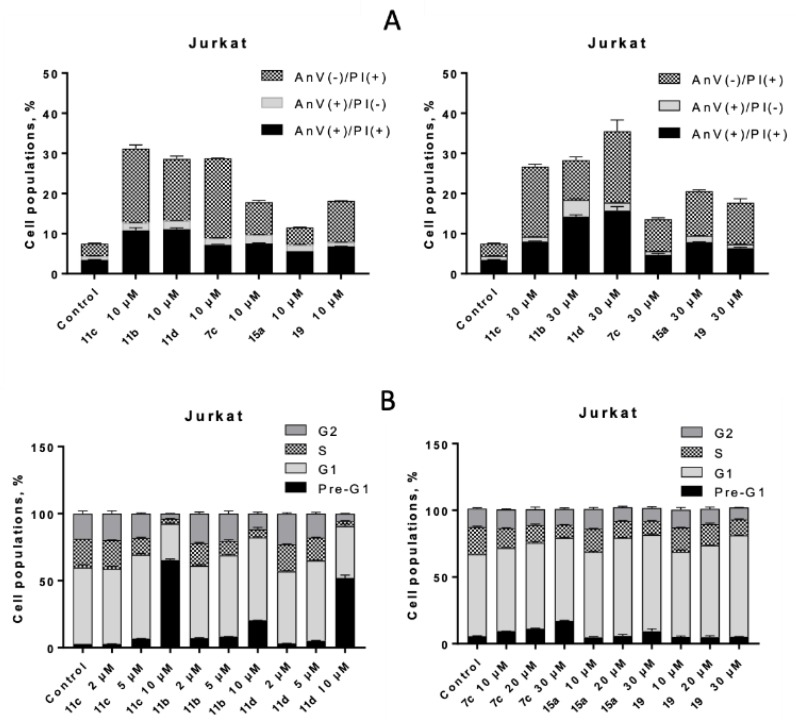
Cell cycle arrest and cell death induction by dual CK2-HDAC inhibitors. (**A**) the impact of the indicated compound doses on the distribution of Jurkat T-leukemia cells in the different phases of the cell cycle was determined by FACS of propidium iodide-stained cells after 24 h of continuous exposure. (**B**) evaluation of the impact of selected dual CK2-HDAC inhibitors (10 µM and 30 µM, 24 h) on phosphatidylserine externalization in Jurkat T-leukemia cells was determined by FACS, using FITC-labeled annexin V and PI staining. One of three experiments delivering comparable data is shown.

To reveal potential mechanisms of cell death induction, selected CK2-HDAC inhibitors were tested in a PI staining assay. All tested compounds demonstrated dose-dependent inhibitory effect on cell cycle progression in human leukemia cells, depleting cell population in S-phase in favor of G_1_ phase (Figure 7). However, this effect was masked for high doses of DMAT-hydroxamate derivatives (30 µM) due to their strong proapoptotic activity, leading to appearance of > 80% cells in pre-G_1_ phase (apoptotic). Thus, lower concentrations of **11b–d** (2 µM and 5 µM) had to be addressed in order to observe their inhibitory effect on cell cycling. On the contrary, compounds with weaker cytotoxic potential (**7c**, **15a** and **19**) possessed mainly cytostatic activity, inhibiting growth of Jurkat T-leukemia cells at G_1_ phase of the cell cycle, while a number of apoptotic cells (pre-G_1_) increased insignificantly even at 30 µM dose (Figure 7). As G_1_/S cell arrest is considered a typical feature of HDAC inhibitors, including SAHA [79], the observed phenomenon is yet another confirmation of HDAC targeting by these novel dual inhibitors in cell culture.

To confirm the pro-apoptotic activity of the studied inhibitors, Annexin V-PI double staining was addressed (Figure 7 down). Compounds with the highest cytotoxic activity (**11b–d**) also led to concentration-dependent twofold increase in number of annexin V-positive (apoptotic) cells compared to derivatives **15a** and **19**, possessing cytostatic activity. On contrary, introduction of the triazole motif in the linker (**7c**) led to a significant decrease in the amount of AnV(+) cells compared to **11d** at 30 µM dose, indicating a negative impact of this group both on cytotoxic and pro-apoptotic activity of **7c**.

#### 2.4.2. Impact of ROS on Circumvention of Cancer Drug Resistance

Rapid development of cancer drug resistance both to traditional and targeted chemotherapies is considered one of the most crucial challenges in modern cancer treatment [80]. Usually, MDR is caused by overexpression of ABC-transporter proteins (namely, P-gp, MRP-1 and BCRP) in tumor cells and CK2 is directly involved in the regulation of their activity [81]. HDAC inhibitors were also shown to induce apoptosis in MDR cell lines [79], thus, combination of pharmacophore’s of CK2 and HDAC inhibitors in one molecule should gradually increase its MDR-circumventing properties. 

Taking into consideration the high abundance of CK2 kinase in leukemia and lymphomas [82], the ability of selected CK2-HDAC dual inhibitors to circumvent MDR was investigated in human leukemia cells of HL-60 line and its doxorubicin-resistant (HL-60/adr, MRP-1 overexpression) and vincristine-resistant (HL-60/vinc, P-gp overexpression) sublines.

**Figure 8:**
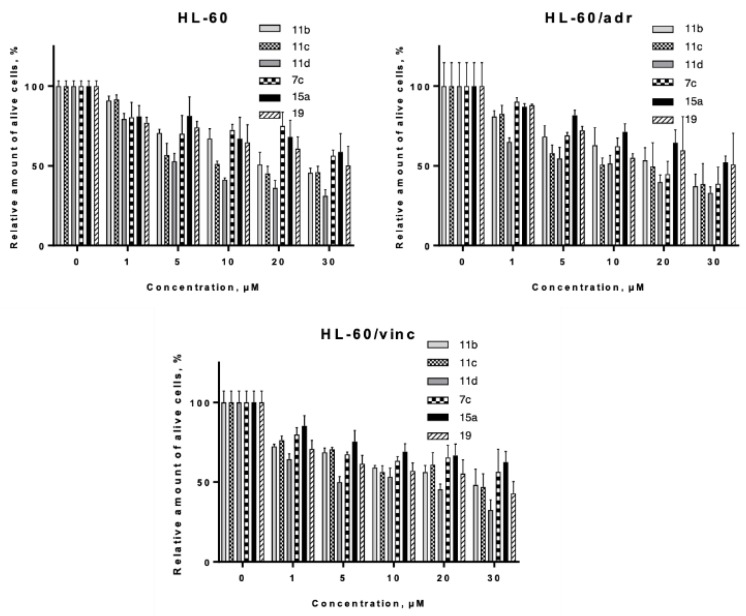
Evaluation of cytotoxic activity of selected CK2-HDAC dual inhibitors towards human leukemia cells of the HL-60 line and its drug-resistant sublines HL-60/adr (MRP-1 overexpression) and HL-60/vinc (P-glycoprotein overexpression). Trypan blue assay, 24 h incubation. Means and SD of at least three experiments in triplicate are shown.

HL-60/wt cells were among the most sensitive to the action of DMAT derived hydroxamates **11b–d**, as revealed by analysis of their LC_50_ values (Table 2, Figure 8). Incubation for 24 h with all the selected inhibitors led to effective killing of both MRP-1 and P-gp overexpressing cells, thus confirming our hypothesis. We observed only slight (~ twofold) decrease of activity of the tested compounds for both P-gp+ and MRP-1 overexpressing cells, except for compound **11d**, which activity was even increased twofold towards the HL-60/vinc cell line. It should be stressed that the aforementioned MDR cell models demonstrated extreme resistance towards traditional anticancer drugs (e.g, 80-fold to doxorubicin for HL-60/adr line and 140-fold to doxorubicin for HL-60/vinc line) [83]. Thus the observed effects of dual CK2-HDAC inhibitors clearly put forward to their potent activity to circumvent MDR in vitro.

To reveal potential mechanisms underlying this phenomenon, we studied the impact of the selected compounds on ROS production in both sensitive and MDR cell lines (Figure S8). It was found that all compounds, except **7c**, had no statistically significant impact on hydrogen peroxide production in all the tested cell lines, as revealed by a DCFDA assay (Figure S8, SI). On the contrary, all the dual inhibitors led to a moderate (30%–50%) increase in superoxide production after 24 h of incubation (revealed by DHE assay), with the exception of compound **7c** on HL-60/vinc cells. As mitochondria are considered the main source of cellular superoxides, the next aim of our studies was to check whether CK2-HDAC inhibitors induce oxidative stress in tumor cells via depolarization of mitochondria, using the JC-1 assay. However, it was technically impossible to measure JC-1 accumulation on HL-60/adr and HL-60/vinc cells, because JC-1 is a substrate for these ABC-transporter proteins and is actively pumped out into extracellular medium.

**Figure 9:**
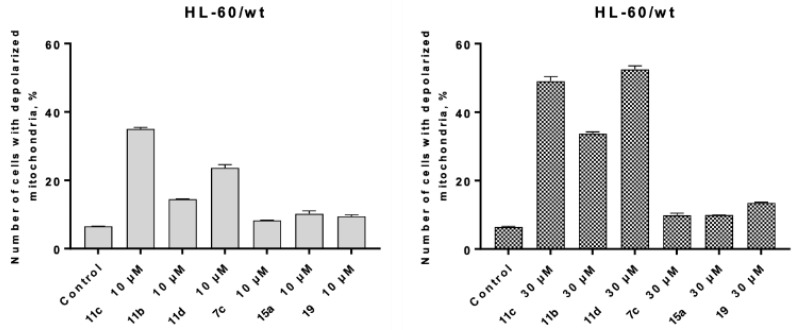
Depolarization of mitochondria in HL-60/wt cells under the action of selected CK2-HDAC dual inhibitors (10 µM and 30 µM, 24 h) was determined by FACS using the Mitochondrial Membrane Potential Assay JC-1. One of three experiments delivering comparable data is shown.

Compounds showing the highest cytotoxic and pro-apoptotic activities in the previous studies, also led to major depolarization of mitochondria in a dose-dependent manner (Figure 9). Notably, **11b** (five methylene groups) led to 14.55% of depolarization of mitochondria at 10 µM, and 34.06% at 30 µM. Compounds with longer aliphatic chains led to even more profound effects (34.73% of depolarized mitochondria at 10 µM of **11c**, and 49.98% at 30 µM). The highest level of depolarized mitochondria was observed for **11d** (53.23%), while in the case of the three other compounds it remained at very low level (9%–10%). This indicates that mitochondria are not involved in apoptosis induction by **7c**, **15a** and **19**.

Specific hydrogen peroxide burst, observed under the action of **7c**, may be explained by the presence of a triazole in its structure. The 3-amino-1,2,4-triazole has been shown to be a specific catalase inhibitor and to lead to major increase in cellular H_2_O_2_ levels [84].

Altogether, circumvention of cancer drug resistance by DMAT derived hydroxamates is accompanied by oxidative stress in tumor cells due to depolarization of mitochondria.

## 3. Materials and Methods 

### 3.1. Chemistry

Unless stated otherwise, purchased starting materials used were high-grade commercial products. NMR spectra were recorded at 400 MHz (^1^*H*) and 100 MHz (^13^*C*) on a Bruker 400-AC magnetic resonance spectrometer at RT in CDCl_3_ [calibrated at 7.26 ppm (^1^*H*) and 77.0 ppm (^13^*C*)] and in DMSO-d_6_ [calibrated at 2.50 ppm (^1^*H*) and 39.5 ppm (^13^*C*)]. Data are presented as follows (^1^*H*): chemical shift (ppm), multiplicity (s singlet, bs broad singlet, d doublet, t triplet, q quartet, m multiplet), coupling constant *J* (Hz) and integration. Data for ^13^*C*-NMR are reported in terms of chemical shifts (ppm). Mass spectra (MS) were determined on a Bruker Esquire 3000 spectrometer (ionizing voltage = 70 eV). Analytical purity of the tested compounds was determined by using an Agilent 1260 Infinity II HPLC system. Melting points (uncorrected) were taken in open-end capillary tubes and were determined on a Stuart Scientific SMP3 apparatus. Thin-layer chromatography (TLC) was run on Merck silica gel 60 F254 plates. Column chromatography was performed using silica gel Merk-60 (230–400 mesh).

**2,4,5,6,7-Pentabromo-1-(prop-2-yn-1-yl)-1*H*-benzo[*d*]imidazole (4a)**

To a solution of **3** [67] (1.95 mmol, 1 g) in acetone (20 mL) in a 20 mL ΜW vessel, K_2_CO_3_ (12.29 μmol, 1.7 g) and 3-bromoprop-1-yne (3.9 mmol, 0.43 mL) were added. The reaction mixture was heated at 110 °C by MW irradiation for 60 minutes. After allowing the reaction to cool to RT, the crude was filtered, removing solid and being the resulting solution concentrated under vacuum and recrystallized in EtOH to afford compound **4a** (906 mg, 84%) as a pale yellow solid (m.p. 200–202 °C). ^1^*H*-NMR (400 MHz, DMSO): δ 5.37 (d, *J* = 2.4 Hz, 2H), 3.63 (t, *J* = 2.4 Hz, 1H). ^13^*C*-NMR (100 MHz, DMSO): δ 142.6, 135.6, 132.2, 123.8, 121.9, 115.6, 106.42, 77.6, 77.5, 37.7.

**2,4,5,6,7-Pentabromo-1-(but-3-yn-1-yl)-1*H*-benzo[*d*]imidazole (4b)**

Following the same procedure as before, starting from **3** [67] (1.95 mmol, 1 g), K_2_CO_3_ (12.29 mmol, 1.7 g) and 4-bromobut-1-yne (3.9 mmol, 0.41 mL), and after recrystallization in EtOH, compound **4b** (437 mg, 40%) was obtained as a yellow solid (m.p. 190–192 °C). ^1^*H*-NMR (400 MHz, DMSO): δ 4.68 (t, *J* = 7.2 Hz, 2H), 2.96 (t, *J* = 2.6 Hz, 1H), 2.75 (td, *J* = 7.2, 2.6 Hz, 2H). ^13^*C*-NMR (100 MHz, DMSO): δ 143.1, 136.7, 132.8, 124.0, 122.0, 115.9, 106.9, 79.8, 74.8, 45.5, 20.7.

**4,5,6,7-tetrabromo-*N*,*N*-dimethyl-1-(prop-2-yn-1-yl)-1*H*-benzo[*d*]imidazol-2-amine (4c)**

To a stirred solution of **4a** (1.19 mmol, 655 mg) in metanol (5 mL), dimethylamine (40% in water, 4.5 mL) was added. The reaction mixture was placed in a sealed tube and refluxed at 110 °C for 48 h. After removing the solvent under reduced pressure, the crude material was purified by column chromatography on silica gel (hexane/EtOAc 8:2) to afford compound **4c** (432 mg, 71%) as white solid (m.p. 194-195 °C). ^1^*H*-NMR (400 MHz, DMSO): δ 4.95 (d, *J* = 2.4 Hz, 2H), 3.53 (t, *J* = 2.4 Hz, 1H), 3.07 (s, 6H). ^13^*C*-NMR (100 MHz, DMSO): δ 161.9, 142.9, 133.4, 120.2, 118.9, 112.5, 105.7, 79.2, 76.9, 41.4, 36.9.

**4,5,6,7-Tetrabromo-1-(but-3-yn-1-yl)-*N*,*N*-dimethyl-1*H*-benzo[*d*]imidazol-2-amine (4d)**

Following the same procedure as before, starting from **4b** (1.24 mmol, 700 mg) and dimethylamine (40% in water, 4.5 mL), and after purification by column chromatography on silica gel (hexane/EtOAc 8:2), compound **4d** (574 mg, 88%) was obtained as white solid (m.p. 190–192 °C). ^1^*H*-NMR (400 MHz, DMSO): δ 4.41 (t, *J* = 6.7 Hz, 2H), 2.99 (s, 6H), 2.78 (t, *J* = 2.6 Hz, 1H), 2.49–2.45 (m, 2H). ^13^*C*-NMR (100 MHz, DMSO): δ 163.1, 143.5, 132.8, 120.2, 119.0, 112.9, 105.9, 79.7, 73.3, 44.5, 41.7, 19.2.

**4-(4-((4,5,6,7-Tetrabromo-2-(dimethylamino)-1*H*-benzo[*d*]imidazol-1-yl)methyl)-1*H*-1,2,3-triazol-1-yl)-*N*-((tetrahydro-2*H*-pyran-2-yl)oxy)butanamide (6a)**

To a solution of alkyne **4c** (0.153 mmol, 35 mg) and azide **5b** [30] (0.168 mmol, 86.6 mg) in DMF (2.5 mL), sodium ascorbate (0.092 mmol, 18 mg), CuSO_4_ × 5 H_2_O (0.05 mmol, 13 mg) and TBTA (0.015 mmol, 1 mg) were added. The resulting mixture was stirred at RT overnight. Then, water was added (10 mL) and the precipitate formed was filtered and purified by column chromatography on silica gel (DCM/MeOH 97.5:2.5) affording compound **6a** (77.7 mg, 70%) as a white solid. ^1^*H*-NMR (400 MHz, CDCl_3_): δ 10.93 (bs, 1H), 7.97 (s, 1H), 5.55 (s, 2H), 4.78 (s, 1H), 4.29 (t, *J* = 6.0 Hz, 2H), 3.96–3.82 (m, 1H), 3.55–3.43 (m, 1H), 2.98 (s, 6H), 2.00-1.89 (m, 4H), 1.70-1.43 (m, 6H). ^13^*C*-NMR (100 MHz, DMSO): δ 168.9, 162.7, 144.6, 143.7, 133.2, 122.3, 121.8, 120.7, 114.4, 105.8, 102.4, 62.6, 49.1, 42.8, 42.4, 29.3, 28.0, 26.0, 25.1, 18.5.

**5-(4-((4,5,6,7-Tetrabromo-2-(dimethylamino)-1*H*-benzo[*d*]imidazol-1-yl)methyl)-1*H*-1,2,3-triazol-1-yl)-*N*-((tetrahydro-2H-pyran-2-yl)oxy)pentanamide (6b)**

Following the same procedure as before, starting from alkyne **4c** (0.291 mmol, 150 mg), azide **5c** [30] (0.265 mmol, 64.2 mg), sodium ascorbate (0.175 mmol, 32 mg), CuSO_4_ × 5 H_2_O (0.087 mmol, 20 mg) and TBTA (0.03 mmol, 1.8 mg), and after purification by column chromatography on silica gel (DCM/MeOH 97.5:2.5), compound **6b** (98.16 mg, 49%) was obtained as a white solid (m.p. 151–153 °C). ^1^*H*- NMR (400 MHz, DMSO): δ 10.89 (bs, 1H), 7.94 (s, 1H), 5.54 (s, 2H), 4.77 (s, 1H), 4.28 (t, *J* = 7.0 Hz, 2H), 3.94–3.79 (m, 1H), 3.53-3.40 (m, 1H), 2.97 (s, 6H), 1.97 (t, *J* = 7.2 Hz, 2H), 1.80–1.17 (m, 10H). ^13^*C*-NMR (100 MHz, DMSO): 165.9, 162.5, 143.4, 142.2, 132.8, 122.9, 120.0, 119.0, 112.9, 105.8, 100.9, 61.3, 45.8, 45.3, 41.6, 32.8, 27.7, 25.9, 24.6, 18.2.

**3-(4-(2-(4,5,6,7-Tetrabromo-2-(dimethylamino)-1*H*-benzo[*d*]imidazol-1-yl)ethyl)-1*H*-1,2,3-triazol-1-yl)-*N*-((tetrahydro-2H-pyran-2-yl)oxy)propanamide (6c)**

Following the same procedure as before, starting from alkyne **4d** (0.284 mmol, 160 mg), azide **5a** [30] (0.258 mmol, 55 mg), sodium ascorbate (0.135 mmol, 30 mg), CuSO_4_ · 5 H_2_O (0.077 mmol, 19 mg) and TBTA (0.0258 mmol, 1.5 mg), and after purification by column chromatography on silica gel (DCM/MeOH 97.5:2.5), compound **6c** (121.4 mg, 63%) was obtained as a white solid (m.p. 140–142 °C). ^1^*H*- NMR (400 MHz, DMSO: δ 11.08 (bs, 1H), 7.71 (s, 1H), 4.76 (s, 1H), 4.59-4.42 (m, 4H), 3.96–3.80 (m, 1H), 3.54–3.40 (m, 1H), 2.92 (s, 6H), 2.87 (t, *J* = 8 Hz, 2H), 2.57 (t, *J* = 6.7 Hz, 2H), 1.73-1.46 (m, 6H). ^13^C- NMR (100 MHz, DMSO): δ 165.9, 162.6, 143.4, 142.2, 132.8, 122.9, 120.0, 119.0, 112.9, 105.8, 100.9, 61.3, 45.8, 45.3, 41.7, 32.8, 27.7, 25.9, 24.6, 18.2.

**4-(4-(2-(4,5,6,7-Tetrabromo-2-(dimethylamino)-1*H*-benzo[*d*]imidazol-1-yl)ethyl)-1*H*-1,2,3-triazol-1-yl)-*N*-((tetrahydro-2*H*-pyran-2-yl)oxy)butanamide (6d)**

Following the same procedure as before, starting from alkyne **4d** (0.283 mmol, 160 mg), azide **5b** [30] (0.258 mmol, 59 mg), sodium ascorbate (0.135 mmol, 30 mg), CuSO_4_ × 5 H_2_O (0.077 mmol, 19 mg) and TBTA (0.0258 mmol, 1.5 mg), and after purification by column chromatography on silica gel (DCM/MeOH 97.5:2.5) compound **6d** (83.4 mg, 43%) was obtained as a white solid (m.p. 125–127 °C). ^1^*H*-NMR (400 MHz, DMSO): δ 10.95 (bs, 1H), 7.73 (s, 1H), 4.81 (s, 1H), 4.53 (t, *J* = 7.2 Hz, 2H), 4.25 (t, *J* = 6.5 Hz, 2H), 3.95-3.83 (m, 1H), 3.54–3.40 (m, 1H), 2.91 (s, 6H), 2.88 (t, *J* = 7.4 Hz, 2H), 2.52 (t, *J* = 1.9 Hz, 2H), 1.96 (bs, 2H), 1.721.43 (m, 6H). ^13^C- NMR (100 MHz, DMSO): δ 168.8, 163.7, 143.7, 132.7, 121.8, 120.3, 113.3, 106.4, 102.0, 62.2, 49.2, 46.8, 41.8, 29.7, 28.0, 26.2, 26.0, 25.0, 18.4.

***N*-Hydroxy-4-(4-((4,5,6,7-tetrabromo-2-(dimethylamino)-1*H*-benzo[*d*]imidazol-1-yl)methyl)-1*H*-1,2,3-triazol-1-yl)butanamide (7a)**

To a solution of **6a** (0.1 mmol, 72 mg) in MeOH (5 mL), a pre-cooled solution of AcCl (1 mmol, 0.07 mL) in MeOH (1 mL) was slowly added over 30 minutes at 0 °C. The reaction mixture was stirred at RT for 15 minutes. Then, NaHCO_3_ (saturated solution) was added until neutral pH. After extraction with EtOAc (3 × 20 mL), the combined organic layers were dried over MgSO_4_. The solvent was evaporated under reduced pressure, and the resulting crude material was purified by column chromatography (DCM/MeOH 98:2) to give **7a** (45 mg, 70%) as a white solid (mp 191–193 °C). ^1^*H*-NMR (400 MHz, DMSO): δ 10.37 (bs, 1H), 8.71 (bs, 1H), 7.99 (s, 1H), 5.55 (s, 2H), 4.28 (t, *J* = 6.7 Hz, 2H), 2.97 (s, 6H), 1.97–1.89 (m, 4H). MS (ESI) m/z 682 [M^+^ + 23]. HPLC purity 99%.

***N*-Hydroxy-5-(4-((4,5,6,7-tetrabromo-2-(dimethylamino)-1*H*-benzo[*d*]imidazol-1-yl)methyl)-1*H*-1,2,3-triazol-1-yl)pentanamide (7b)**

Following the same procedure as before, starting from **6b** (0.21 mmol, 158 mg) and AcCl (2.1 mmol, 0.15 mL), and after purification by column chromatography (DCM/MeOH 98:2), **7b** (14.8 mg, 11%) was obtained as a white solid (mp 203–205 °C). ^1^*H*- NMR (400 MHz, DMSO): δ 10.33 (bs, 1H), 8.66 (bs, 1H), 7.95 (s, 1H), 5.55 (s, 2H), 4.27 (t, *J* = 7.0 Hz, 2H), 2.98 (s, 6H), 1.93 (t, *J* = 7.3 Hz, 2H), 1.74–1.67 (m, 2H), 1.39–1.32 (m, 2H). MS (ESI) m/z 696 [M^+^ + 23], 674 [M^+^ + 1]. HPLC purity 95%.

***N*-Hydroxy-3-(4-(2-(4,5,6,7-tetrabromo-2-(dimethylamino)-1*H*-benzo[*d*]imidazol-1-yl)ethyl)-1*H*-1,2,3-triazol-1-yl)propanamide (7c)**

Following the same procedure as before, starting from **6c** (0.16 mmol, 121.4 mg) and AcCl (1.6 mmol, 0.11 mL) and after purification by column chromatography (DCM/MeOH 98:2), **7c** (18.9 mg, 18%) was obtained as a white solid (mp 168-170 °C). ^1^*H*- NMR (400 MHz, DMSO): δ 10.49 (bs, 1H), 8.83 (bs, 1H), 7.73 (s, 1H), 4.53–4.45 (m, 4H), 2.92 (s, 6H), 2.87 (t, *J* = 7.3 Hz, 2H), 2.56-2.52 (m, 2H). MS (ESI) m/z 682 [M^+^ + 23], 660 [M^+^ + 1]. HPLC purity 95%.

***N*-Hydroxy-4-(4-(2-(4,5,6,7-tetrabromo-2-(dimethylamino)-1*H*-benzo[*d*]imidazol-1-yl)ethyl)-1*H*-1,2,3-triazol-1-yl)butanamide (7d)**

Following the same procedure as before, starting from **6d** (0.1 mmol, 79 mg) and AcCl (1 mmol, 0.07 mL), and after purification by column chromatography (DCM/MeOH 98:2), **7d** (31 mg, 47%) was obtained as a white solid (159–161 °C). ^1^*H*- NMR (400 MHz, DMSO): δ 10.39 (bs, 1H), 8.73 (bs, 1H), 7.74 (s, 1H), 4.53 (“t”, 2H), 4.23–4.27 (m, 2H), 2.91 (s, 6H), 2.89 (“t”, 2H), 2.60 (m, under DMSO, 4H). MS (ESI) m/z 696 [M^+^ + 23], 674 [M^+^ + 1]. HPLC purity 98%.

**Methyl 5-(4,5,6,7-tetrabromo-2-(dimethylamino)-1*H*-benzo[*d*]imidazol-1-yl)pentanoate (8a).**

**DMAT** [74] (0.5 mmol, 238 mg), K_2_CO_3_ (3.5 mmol, 0.48 g), methyl 5-bromopentanoate (2 mmol, 0.39 g) and acetone (10 mL) were placed in a sealed tube and the mixture was heated at 80 °C for 72 h. After removing the solvent under reduced pressure, the crude was purified by column chromatography on silica gel (hexane/EtOAc 8:2) affording compound **8a** (183 mg, 62%) as a colorless oil. ^1^*H*- NMR (400 MHz, CDCl_3_): δ 4.27 (t, *J* = 7.2 Hz, 2H), 3.64 (s, 3H), 3.06 (s, 6H), 2.28 (t, *J* = 7.3 Hz, 2H), 1.63–1.60 (m, 2H), 1.42–1.49 (m, 2H). ^13^*C*-NMR (100 MHz, CDCl_3_): δ 173.4, 162.9, 143.5, 132.7, 121.2, 120.5, 114.1, 105.9, 51.6, 46.1, 42.4, 33.3, 29.7, 21.4.

**Ethyl 6-(4,5,6,7-tetrabromo-2-(dimethylamino)-1*H*-benzo[*d*]imidazol-1-yl)hexanoate (8b)**

Following the same procedure as before, and starting from **DMAT** [74] (0.5 mmol, 238 mg), K_2_CO_3_ (3.5 mmol, 0.48 g) and ethyl 6-bromohexanoate (2 mmol, 0.35 mL), **8b** (222 mg, 72%) was obtained as a colorless oil. ^1^*H*- NMR (400 MHz, CDCl_3_): δ 4.26–4.22 (m, 2H), 4.10 (q, *J* = 7.1 Hz, 2H), 3.02 (s, 6H), 2.23 (t, *J* = 7.4 Hz, 2H), 1.62–1.54 (m, 4H), 1.23 (t, *J* = 7.1 Hz, 3H), 1.18–1.13 (m, 2H). ^13^*C*-NMR (100 MHz, CDCl_3_): δ 173.3, 162.9, 143.6, 132.8, 121.0, 120.3, 114.1, 105.9, 60.3, 46.3, 42.3, 34.0, 29.9, 25.6, 24.3, 14.2.

**Ethyl 7-(4,5,6,7-tetrabromo-2-(dimethylamino)-1*H*-benzo[*d*]imidazol-1-yl)heptanoate (8c)**

Following the same procedure as before, and starting from **DMAT** [74] (0.5 mmol, 238 mg), K_2_CO_3_ (3.5 mmol, 0.48 g) and ethyl 7-bromoheptanoate (2 mmol, 0.39 mL), **8c** (246 mg, 78%) was obtained as a colorless oil. ^1^*H*- NMR (400 MHz, CDCl_3_): δ 4.24 (t, *J* = 7.4 Hz, 2H), 4.11 (q, *J* = 7.1 Hz, 2H), 3.03 (s, 6H), 2.24 (t, *J* = 7.4 Hz, 2H), 1.60–1.52 (m, 4H), 1.32–1.22 (m, 5H), 1.18–1.13 (m, 2H). ^13^*C*-NMR (100 MHz, CDCl_3_): δ 173.5, 163.0, 143.6, 132.8, 120.9, 120.3, 114.1, 105.9, 60.2, 46.5, 42.3, 34.1, 30.1, 28.5, 25.8, 24.7, 14.2.

**Methyl 8-(4,5,6,7-tetrabromo-2-(dimethylamino)-1*H*-benzo[*d*]imidazol-1-yl)octanoate (8d)**

Following the same procedure as before, and starting from **DMAT** [74] (0.42 mmol, 201 mg), K_2_CO_3_ (3 mmol, 0.41 g) and methyl 8-bromooctanoate (2 mmol, 0.39 g), **8d** (177 mg, 67%) was obtained as a colorless oil. ^1^*H*- NMR (400 MHz, CDCl_3_): δ 4.23 (t, *J* = 7.2 Hz, 2H), 3.66 (s, 3H), 3.04 (s, 6H), 2.27 (t, *J* = 7.5 Hz, 2H), 1.59–1.53 (m, 4H), 1.28–1.24 (m, 4H), 1.15–1.11 (m, 2H).

**5-(4,5,6,7-Tetrabromo-2-(dimethylamino)-1*H*-benzo[*d*]imidazol-1-yl)pentanoic acid (9a)**

To a solution of **8a** (0.27 mmol, 160 mg) in THF/H_2_O (2.5 mL/1 mL), LiOH (2 mL, 2.5N in water) was added and the reaction mixture was stirred at RT for 2 h. After removing solvents under reduced pressure, H_2_O (20 mL) was added and EtOAc (20 mL) to wash. The aqueous phase was acidified with HCl (1 M) and extracted with EtOAc (2 × 20 mL). Combined organic layers were washed with brine, dried over MgSO_4_, filtered and the solvent was evaporated under reduced pressure to afford compound **9a** (116 mg, 75%) as a white oil. ^1^*H*-NMR (400 MHz, DMSO): δ 12.01 (bs, 1H), 4.26 (t, *J* = 7.1 Hz, 2H), 2.98 (s, 6H), 2.17 (t, *J* = 7.3 Hz, 2H), 1.57–1.50 (m, 2H), 1.28–1.23 (m, 2H). ^13^*C*-NMR (100 MHz, DMSO): δ 174.1, 162.6, 143.3, 132.9, 120.0, 119.1, 112.9, 106.0, 45.9, 41.8, 32.9, 29.1, 21.0.

**6-(4,5,6,7-Tetrabromo-2-(dimethylamino)-1*H*-benzo[*d*]imidazol-1-yl)hexanoic acid (9b)**

Following the same procedure as before, and starting from **8b** (0.28 mmol, 172 mg) and LiOH (2 mL, 2.5N in water), **9b** (129 mg, 79%) was obtained as a white solid. ^1^*H*-NMR (400 MHz, DMSO): δ 4.24 (t, *J* = 7.3 Hz, 2H), 2.98 (s, 6H), 2.12 (t, *J* = 7.3 Hz, 2H), 1.53–1.42 (m, 4H), 1.11–1.07 (m, 2H). ^13^*C*- NMR (100 MHz, DMSO): δ 174.3, 162.8, 143.3, 132.9, 119.9, 119.0, 112.9, 105.8, 46.0, 41.8, 33.6, 29.4, 25.0, 24.0.

**7-(4,5,6,7-Tetrabromo-2-(dimethylamino)-1*H*-benzo[*d*]imidazol-1-yl)heptanoic acid (9c)**

Following the same procedure as before, and starting from **8c** (0.38 mmol, 243 mg) and LiOH (2 mL, 2.5N in water), **9c** (201 mg, 88%) was obtained as a white solid. ^1^*H*- NMR (400 MHz, DMSO): δ 4.24 (t, *J* = 7.2 Hz, 2H), 2.98 (s, 6H), 2.15–2.11 (m, 2H), 1.53–1.45 (m, 2H), 1.44–1.36 (m, 2H), 1.25–1.17 (m, 2H), 1.09–1.02 (m, 2H). ^13^*C*- NMR (100 MHz, DMSO): δ 174.4, 163.0, 143.4, 133.0, 120.0, 119.0, 112.9, 105.9, 46.1, 41.8, 33.5, 29.5, 27.9, 25.2, 24.3.

**8-(4,5,6,7-Tetrabromo-2-(dimethylamino)-1*H*-benzo[*d*]imidazol-1-yl)octanoic acid (9d)**

Following the same procedure as before, and starting from **8d** (0.49 mmol, 312 mg) and LiOH (4.6 mL, 2.5N in water), **9d** (183 mg, 60%) was obtained as a colorless oil. ^1^*H*- NMR (400 MHz, DMSO): δ 11.96 (bs, 1H), 4.24 (t, *J* = 7.2 Hz, 2H), 2.98 (s, 6H), 2.15 (t, *J* = 7.3 Hz, 2H), 1.52–1.48 (m, 2H), 1.43–1.40 (m, 2H), 1.20–1.15 (m, 4H), 1.05–1.02 (m, 2H).

**5-(4,5,6,7-Tetrabromo-2-(dimethylamino)-1*H*-benzo[*d*]imidazol-1-yl)-*N*-((tetrahydro-2*H*-pyran-2-yl)oxy)pentanamide (10a)**

To a solution of **9a** (0.42 mmol, 242 mg) in DMF (10 mL) were successively added HOBt (0.5 mmol, 78 mg), NMM (1.26 mmol, 0.14 mL), NH_2_OTHP (0.84 mmol, 98 mg) and EDCI (0.59 mmol, 0.1 mL). The resulting mixture was stirred at RT overnight. H_2_O (20 mL) and EtOAc (3 × 20 mL) were added. After extractions, combined organic layers were washed with brine, dried over MgSO_4_, filtered and evaporated under reduced pressure. The crude obtained was purified by column chromatography on silica gel (EtOAc/hexane 8:2) affording compound **10a** (136 mg, 50%) as a white solid. ^1^*H*-NMR (400 MHz, DMSO): δ 10.86 (bs, 1H), 4.71 (s, 1H), 4.24 (m, 2H), 3.84 (t, *J* = 10.0 Hz, 1H), 3.40 (m, 1H), 2.98 (s, 6H), 1.93 (t, *J* = 7.1 Hz, 2H), 1.64–1.47 (m, 8H), 1.30–1.26 (m, 2H). ^13^*C*- NMR (100 MHz, DMSO): δ 168.6, 162.9, 143.4, 132.9, 120.0, 119.0, 112.9, 106.0, 100.8, 61.3, 46.0, 41.8, 31.6, 29.2, 27.7, 24.7, 21.6, 18.3.

**6-(4,5,6,7-Tetrabromo-2-(dimethylamino)-1*H*-benzo[*d*]imidazol-1-yl)-*N*-((tetrahydro-2*H*-pyran-2-yl)oxy)hexanamide (10b)**

Following the same procedure as before, and starting from **9b** (0.32 mmol, 193 mg), HOBt (0.38 mmol, 58 mg), NMM (0.96 mmol, 0.11 mL), NH_2_OTHP (0.64 mmol, 75 mg) and EDCI (0.44 mmol, 0.08 mL), **10b** (61 mg, 28%) was obtained as a white solid. ^1^*H*- NMR (400 MHz, CDCl_3_): δ 8.25 (bs, 1H), 4.91 (s, 1H), 4.23 (t, *J* = 7.2 Hz, 2H), 3.93–3.89 (m, 1H), 3.63–3.60 (m, 1H), 3.02 (s, 6H), 2.09–1.98 (m, 2H), 1.81–1.76 (m, 2H), 1.67–1.55 (m, 8H), 1.19–1.14 (m, 2H). ^13^*C*- NMR (100 MHz, CDCl_3_): δ 170.0, 163.0, 143.6, 132.8, 121.0, 120.3, 114.0, 105.9, 102.5, 62.5, 46.3, 42.3, 32.9, 29.9, 27.9, 25.6, 24.9, 24.6, 18.5.

**7-(4,5,6,7-Tetrabromo-2-(dimethylamino)-1*H*-benzo[d]imidazol-1-yl)-*N*-((tetrahydro-2H-pyran-2-yl)oxy)heptanamide (10c)**

Following the same procedure as before, and starting from **9c** (0.51 mmol, 313 mg), HOBt (0.61 mmol, 91 mg), NMM (1.53 mmol, 0.16 mL), NH_2_OTHP (1.02 mmol, 117 mg) and EDCI (0.71 mmol, 0.12 mL), **10c** was obtained (182 mg, 51%) as a white solid. ^1^*H*- NMR (400 MHz, CDCl_3_): δ 8.19 (bs, 1H), 4.92 (s, 1H), 4.23 (t, *J* = 7.2 Hz, 2H), 3.95–3.89 (m, 1H), 3.63–3.60 (m, 1H), 3.03 (s, 6H), 2.05 (m, 2H), 1.80–1.77 (m, 2H), 1.62–1.52 (m, 8H), 1.30–1.25 (m, 2H), 1.17–1.12 (m, 2H). ^13^*C*- NMR (100 MHz, CDCl_3_): δ 170.0, 163.0, 143.6, 132.9, 121.0, 120.3, 114.0, 105.9, 102.5, 62.6, 46.5, 42.4, 42.3, 33.1, 30.1, 28.5, 28.0, 25.9, 25.0, 18.5.

**8-(4,5,6,7-Tetrabromo-2-(dimethylamino)-1*H*-benzo[*d*]imidazol-1-yl)-*N*-((tetrahydro-2H-pyran-2-yl)oxy)octanamide (10d)**

Following the same procedure as before, and starting from **9d** (0.29 mmol, 183 mg), HOBt (0.34 mmol, 53 mg), NMM (0.87 mmol, 0.09 mL), NH_2_OTHP (0.58 mmol, 89 mg) and EDCI (0.39 mmol, 0.07 mL), **10d** (32.4 mg, 16%) was obtained as a white solid. ^1^*H*- NMR (400 MHz, CDCl_3_): δ 4.92 (s, 1H), 4.22–4.17 (m, 2H), 3.92–3.87 (m, 1H), 3.56–3.54 (m, 1H), 3.03 (s, 6H), 2.09–2.06 (m, 2H), 1.79–1.69 (m, 2H), 1.57–1.51 (m, 10H), 1.18–1.14 (m, 2H), 1.08–1.13 (m, 2H).

***N*-Hydroxy-5-(4,5,6,7-tetrabromo-2-(dimethylamino)-1*H*-benzo[*d*]imidazol-1-yl)pentanamide (11a)**

Following the same procedure that we used for the synthesis of **7a**, starting from **10a** (0.21 mmol, 136 mg) and AcCl (2.1 mmol, 0.15 mL), and after purification by column chromatography (EtOAc/Hexane 4:1), **11a** (36 mg, 30%) was obtained as a white solid (m.p.168 - 170 °C). ^1^*H*- NMR (400 MHz, DMSO): δ 10.30 (bs, 1H), 8.67 (bs, 1H), 4.25 (t, *J* = 7.0 Hz, 2H), 2.98 (s, 6H), 1.89 (t, *J* = 7.3 Hz, 2H), 1.50–1.46 (m, 2H), 1.29–1.25 (m, 2H). MS (ESI) m/z 615 [M^+^ + 23], 593 [M^+^ + 1]. HPLC purity 98%.

***N*-Hydroxy-6-(4,5,6,7-tetrabromo-2-(dimethylamino)-1*H*-benzo[*d*]imidazol-1-yl)hexanamide (11b)**

Following the same procedure that we used for the synthesis of **7a**, starting from **10b** (0.09 mmol, 61 mg), and AcCl (0.9 mmol, 0.07 mL) and after purification by column chromatography (EtOAc/Hexane 4:1), **11b** (38 mg, 70%) was obtained as a white solid (m.p. 167–169 °C). ^1^*H*- NMR (400 MHz, DMSO): δ 10.29 (bs, 1H), 8.64 (bs, 1H), 4.23 (t, *J* = 7.1 Hz, 2H), 2.99 (s, 6H), 1.87 (t, *J* = 7.3 Hz, 2H), 1.50–1.44 (m, 4H), 1.08–1.06 (m, 2H). MS (ESI) m/z 629 [M^+^ + 23], 607 [M^+^ + 1]. HPLC purity 97%.

***N*-Hydroxy-7-(4,5,6,7-tetrabromo-2-(dimethylamino)-1*H*-benzo[*d*]imidazol-1-yl)heptanamide (11c)**

Following the same procedure that we used for the synthesis of **7a**, starting from **10c** (0.25 mmol, 180 mg) and AcCl (2.5 mmol, 0.18 mL), and after purification by column chromatography (DCM/MeOH 98:2), **11c** (50 mg, 33%) was obtained as a white solid (m.p. 120-122 °C). ^1^*H*- NMR (400 MHz, DMSO): δ 10.3 (bs, 1H), 8.62 (bs, 1H), 4.23 (t, *J* = 7.3 Hz, 2H), 2.99 (s, 6H), 1.89 (t, *J* = 7.2 Hz, 2H), 1.51–1.48 (m, 2H), 1.42–1.38 (m, 2H), 1.21–1.17 (m, 2H), 1.08–1.04 (m, 2H). MS (ESI) m/z 643 [M^+^ + 23], 621 [M^+^+ 1]. HPLC purity 98%.

***N*-Hydroxy-8-(4,5,6,7-tetrabromo-2-(dimethylamino)-1*H*-benzo[*d*]imidazol-1-yl)octanamide (11d)**

Following the same procedure that we used for the synthesis of **7a**, starting from **10d** (0.12 mmol, 89.9 mg) and AcCl (1.2 mmol, 0.08 mL), and after purification by column chromatography (DCM/MeOH 98:2), **11d** (19 mg, 25%) was obtained as a white solid (m.p. 118 °C). ^1^*H*- NMR (400 MHz, DMSO): δ 10.3 (bs, 1H), 8.64 (bs, 1H), 4.24 (t, *J* = 7.3 Hz, 2H), 2.99 (s, 6H), 1.89 (t, *J* = 7.3 Hz, 2H), 1.51–1.48 (m, 2H), 1.43–1.40 (m, 2H), 1.19–1.13 (m, 4H), 1.06–1.02 (m, 2H). MS (ESI) m/z 657 [M^+^ + 23], 635 [M^+^+ 1]. HPLC purity 95%.

**Methyl 4-((tetrabromo-2*H*-benzo[*d*][1,2,3]triazol-2-yl)methyl)benzoate (12a) and methyl 4-((tetrabromo-1*H*-benzo[*d*][1,2,3]triazol-1-yl)methyl)benzoate (12b)**

A solution of **TBB** [73] (200 mg, 0.45 mmol), K_2_CO_3_ (9 eq) and methyl 4-(bromomethyl)benzoate (105 mg, 0.65 mmol) in acetone (5 mL) was irradiated under MW conditions at 150 °C for one minute. The solvent was removed under vacuum and the crude was solved in EtOAc. The organic phase was washed with brine and H_2_O, dried over MgSO_4_, filtered and evaporated. The crude material was purified by column chromatography on silica gel (hexane/DCM 35:65), giving **12a** (215 mg, 80%) and **12b** (50 mg, 18%). For **12a**: ^1^*H*-NMR (400 MHz, DMSO) δ 7.98 (d, *J* = 8.3 Hz, 2H), 7.51 (d, *J* = 8.3 Hz, 2H), 6.19 (s, 2H), 3.84 (s, 3H). For 1**2b**: ^1^*H*-NMR (400 MHz, DMSO) δ 7.84 (d, *J* = 8.4 Hz, 2H), 7.23 (d, *J* = 8.3 Hz, 2H), 6.30 (s, 2H), 3.83 (s, 3H).

**4-((Tetrabromo-2*H*-benzo[*d*][1,2,3]triazol-2-yl)methyl)benzoic acid (13a)**

A solution of **12a** (50 mg, 0.09 mmol) in 1:1 THF:NaOH 1M (40 eq) was stirred at RT till completion of the reaction (TLC). The crude was then washed with EtOAc and the aqueous phase was acidified with HCl 3M. The precipitate obtained was filtered and used in the next reaction without further purification. ^1^*H*-NMR (400 MHz, DMSO) δ 13.06 (bs, 1H), 7.95 (d, *J* = 8.2 Hz, 2H), 7.49 (d, *J* = 8.2 Hz, 2H), 6.17 (s, 2H). ^13^C NMR (100 MHz, DMSO) δ 166.8, 142.9, 138.9, 131.0, 129.8, 128.4, 125.9, 113.7, 59.8.

**4-((Tetrabromo-1*H*-benzo[*d*][1,2,3]triazol-1-yl)methyl)benzoic acid (13b)**

Following the same procedure as before and starting from **12b** (70 mg, 0.11 mmol), **13b** (60 mg, 98%) was obtained as a white solid. ^1^H-NMR (400 MHz, DMSO) δ 7.89 (d, *J* = 8.4 Hz, 2H), 7.21 (d, *J* = 8.4 Hz, 2H), 6.29 (s, 2H).

**4-((Tetrabromo-2*H*-benzo[*d*][1,2,3]triazol-2-yl)methyl)-*N*-((tetrahydro-2*H*-pyran-2-yl)oxy)benzamide (14a)**

Following the same procedure that was used for the synthesis of **10a** and starting from **13a** (0.21 mmol, 188 mg), HOBt (0.25 mmol, 39 mg), NMM (0.63 mmol, 0.07 mL), NH_2_OTHP (0.42 mmol, 49 mg) and EDCI (0.29 mmol, 0.05 mL), **14a** (60 mg, 98%) was obtained, after column chromatography on silica gel (EtOAc/hexane 3:7), as a white solid. ^1^*H*-NMR (400 MHz, DMSO) δ 8.71 (bs, 1H), 7.76 (d, *J* = 8.3 Hz, 2H), 7.51 (d, *J* = 8.3 Hz, 2H), 5.92 (s, 2H), 5.06 (bs, 1H), 4.03–3.89 (m, 1H), 3.69–3.57 (m, 1H), 1.93–1.57 (m, 6H).

**4-((Tetrabromo-1*H*-benzo[*d*][1,2,3]triazol-1-yl)methyl)-*N*-((tetrahydro-2*H*-pyran-2-yl)oxy)benzamide (14b)**

Following the same procedure that was used for the synthesis of **10a** and starting from **13b** (0.11 mmol, 60 mg), HOBt (0.12 mmol, 20 mg), NMM (0.31 mmol, 0.04 mL), NH_2_OTHP (0.21 mmol, 25 mg) and EDCI (0.15 mmol, 0.03 mL), **14b** (60 mg, 98%) was obtained, after column chromatography on silica gel (EtOAc/hexane 2:1 to 1:1), as a white solid. ^1^*H*-NMR (400 MHz, DMSO) δ 9.03 (bs, 1H), 7.71 (d, *J* = 8.2 Hz, 2H), 7.15 (d, *J* = 8.0 Hz, 2H), 6.18 (s, 2H), 5.04 (bs, 1H), 3.98–3.90 (m, 1H), 3.72–3.51 (m, 1H), 1.91–1.47 (m, 6H).

***N*-Hydroxy-4-((tetrabromo-2*H*-benzo[*d*][1,2,3]triazol-2-yl)methyl)benzamide (15a)**

Following the same procedure that we used for the synthesis of **7a**, starting from **14a** (0.12 mmol, 80 mg) and AcCl (1.2 mmol, 0.09 mL), crude reaction was obtained. A precipitate formed after addition of Et_2_O was filtered and solved in DCM/MeOH (5%) and precipitated again with hexane. The solid was filtered, giving **15a** (70 mg, quantitative conversion) as a white solid (m.p. 218–220 °C). ^1^*H*-NMR (400 MHz, DMSO) δ 11.23 (bs, 1H), 9.07 (bs, 1H), 7.75 (d, *J* = 8.3 Hz, 2H), 7.46 (d, *J* = 8.3 Hz, 2H), 6.12 (s, 2H). MS (ESI) m/z 584.6 [M^+^+1]. HPLC purity 94%.

***N*-Hydroxy-4-((tetrabromo-1*H*-benzo[*d*][1,2,3]triazol-1-yl)methyl)benzamide (15b)**

Following the same procedure as before starting from **14b** (0.067 mmol, 50 mg,), and AcCl (0.67 mmol, 0.05 mL), **15b** (30 mg, 77%) was obtained as a white solid (m.p. 139-142 °C). ^1^*H*-NMR (400 MHz, DMSO) δ 11.22 (bs, 1H), 7.70 (d, *J* = 8.4 Hz, 2H), 7.18 (d, *J* = 8.4 Hz, 2H), 6.25 (s, 2H). MS (ESI) m/z 584.6 [M^+^+1]. HPLC purity 97%.

**Methyl 4-((4,5,6,7-tetrabromo-2-(dimethylamino)-1*H*-benzo[d]imidazol-1-yl)methyl)benzoate (16)**

A mixture of **DMAT** [74] (150 mg, 0.314 mmol), methyl 4-(bromomethyl)benzoate (72.22 mg, 0.314 mmol) and K_2_CO_3_ (130 mg, 0.942 mmol) in acetone (2.5 mL) was refluxed for 48 h. The reaction mixture was evaporated to dryness, diluted in EtOAc (10 mL) and washed with brine (2×10 mL). The crude material was dried with MgSO_4_, filtered, evaporated to dryness and purified by column chromatography (hexane/EtOAc 9:1 to 1:1) to give **16** (182 mg, 93%) as a white solid (m.p. 106–108 °C). ^1^H-NMR (400 MHz, CDCl_3_) δ 7.98 (d, *J* = 8.4 Hz, 2H), 7.07 (d, *J* = 8.4 Hz, 2H), 5.55 (s, 2H), 3.89 (s, 3H), 2.97 (s, 6H).

**4-((4,5,6,7-Tetrabromo-2-(dimethylamino)-1*H*-benzo[*d*]imidazol-1-yl)methyl)-N-((tetrahydro-2H-pyran-2-yl)oxy)benzamide (18)**

Following the same procedure that we used for the synthesis of **14a**, and starting from **16** (170 mg, 0.27 mmol), **17** (145 mg, 88%) was obtained as an oil which was used in the next step without further purification. **17** (140 mg, 0.23 mmol) was treated as described for **15a**, and after purification by column chromatography on silica gel (hexane/EtOAc 8:2 to 1:1) gave **18** (100 mg, 61%) as a white solid. ^1^H-NMR (400 MHz, CDCl_3_) δ 8.84 (s, 1H), 7.69 (d, *J* = 8.2 Hz, 2H), 7.06 (d, *J* = 8.2 Hz, 2H), 5.53 (s, 2H), 5.05 (s, 1H), 3.99–3.82 (m, 1H), 3.70–3.52 (m, 1H), 2.96 (s, 6H), 1.92–1.56 (m, 6H).

***N*-Hydroxy-4-((4,5,6,7-tetrabromo-2-(dimethylamino)-1*H*-benzo[*d*]imidazol-1-yl)methyl)benzamide (19)**

Following the same procedure that we used for the synthesis of **7a**, and starting from **18** (90 mg, 0.126 mmol), **19** (60 mg, 79%) was obtained as a white solid (m.p. 210–213 °C). ^1^H-NMR (400 MHz, DMSO) δ 7.67 (d, *J* = 8.3 Hz, 2H), 7.07 (d, *J* = 8.3 Hz, 2H), 5.57 (s, 2H), 2.94 (s, 6H). MS (ESI) m/z 648.7 [M^+^+23]. HPLC purity 95%.

### 3.2. Molecular Modelling

To date, 158 crystal structures of the CK2 alpha catalytic subunit have been deposited in the PDB database (UniProt Entry NX_P68400 Human CK2 alpha). Four of them (3RPS, 4kWP, 5CQW, 5CQU) are bound to TBB derivatives, including compound JRJ that is the most similar to our set of molecules. Interestingly, JRJ is crystallized in two different conformations: in 5CQU the flexible chain points to the ATP phosphate-binding region, whereas in 5CQW it points to the α-D helix. Of these two 3D structures, we selected 5CQU as the protein shows a more open G-loop compared to 5CQW. For HDAC1, despite its biological interest, only two 3D-structures have been deposited in the PDB: 4BKX and 5ICN (UniProt Entry Q13547 Human Histone deacetylase 1). Of those, PDB code 5ICN was selected due to the presence of a hydroxamic derivative bound to the holo structure [85]. For HDAC6 there is only one available crystal structure, PDB code 5EDU (UniProt entry Q9UBN7 human histone deacetylase 6). This structure presents both domains (histone deacetylase I & II) in the holo conformation bound to the inhibitor Trichostatin A [86]. The hydroxamic-zinc interaction in metalloproteinases has been intensively studied and it has been established that upon binding, the pKa of the hydroxamic acid decreases, resulting in the spontaneous deprotonation of the terminal hydroxyl group, transferring the proton to the histidine (His 140 in HDAC1 and His610 in HDAC6) within the active site [87]. All receptors were processed with the Protein Preparation Wizard module of Maestro [88], but taking into account the HDAC reactivity in the presence of hydroxamic acids, both HDAC1 and HDAC1 were prepared with protonated histidines in positions 140 and 610, respectively.

The Glide Grid (Glide, Schrödinger, LLC, New York, NY, 2018) module was used to calculate the grid-box with a 20 Å length, using as box center the ligands JRJ and TSN for CK2 and HDAC6, respectively; and, the catalytic Zn for HDAC1, applying the versatile metal binding restriction to the zinc ion in both HDACs. Note must be taken that when using the ligand as box center, the program automatically deletes the ligand for the grid generation. The set of described ligands were prepared with the LigPrep module of Maestro (S. LigPrep, Schrödinger Release 2018-2, LLC, New York) considering a pH of 7 ± 4, and to obtain all possible hydroxamic protonation states and the metal binding site identification was also used. Ligands with a protonated hydroxamic acid were used for docking on CK2 whereas ligands with the deprotonated hydroxamate were docked in HDAC1 and HDAC6. The docking calculations were performed with Glide using the XP algorithm [89], applying van der Waals radii scaling factor of 1.0/0.8 and establishing halogen atoms as acceptors. Finally, visual inspection was applied to select the most plausible ligand orientation within the enzyme, which was further used for MD simulations.

For the MD simulations of the CK2-ligand complexes, all ligands were prepared as follows: the geometry optimization and charge distributions were calculated quantum mechanically (RHF/6-31+G**) with Gaussian 09 Revision A.1 (Gaussian, Inc., Wallingford, CT).The general AMBER force field 2 (GAFF2) was used to assign bonded and nonbonded parameters [90].

For HDAC1 and HDAC6-ligand complexes, binding metal sites and ligands were parametrized with the MCPB module embedded into the MTK++ software package in AMBER16 [91], as this methodology has been widely used to facilitate the modeling of metal effects on metalloproteins [92]. The optimized zinc and potassium metal coordination spheres were obtained by geometry optimization in the gas phase using Gaussian09 at B3LYP/6-31G* level, getting the equilibrium values of bond lengths, angles and force constants of the atoms bound to the metal.

Classical molecular dynamics (MD) simulations were performed on all complexes, with the AMBER16 program (http://ambermd.org/). The classic ff14SB AMBER force field [93] for the protein parametrization was applied together with previously assigned active site and ligand parameters. After that, systems were minimized at vacuum, to release possible undesired interactions. Complexes were then embedded in a TIP3P water octahedron of approximately 10 000 to 13 000 water molecules. System neutrality was achieved in all systems by adding sodium or chlorine ions when necessary. Water molecules and counter ions were minimized and then the embedded system was heated to 300 K for 25 ps at constant volume, keeping the protein restrained to initial positions using quadratic harmonic restraints with a constant force of 50 kcal mol^−1^Å^−2^. In all systems, the hydrogen bond lengths were kept at their equilibrium distance by means of the SHAKE algorithm [94]. Atom pair distance cutoffs were applied at 10.0 Å to compute the van der Waals interactions, while long-range electrostatics were computed by means of particle-mesh Ewald (PME) method [95]. Finally, MD simulation production was performed up to 20 ns using the thermostat NPT ensemble at 300 K, generating snapshots each 20 ps for further analysis. The trajectories of all complexes were collected and analyzed by the cpptraj module of AMBER16 [96] in order to obtain the root mean square deviation (RMSD) value of the atomic positions of the ligands.

### 3.3. Biological Assays

In Vitro HDAC1 and HDAC6 inhibitions were performed using Fluorometric Drug Discovery Assay Kits from Enzo Life Sciences, Inc. The reactions were prepared in HDAC assay buffer (50 mM Tris-HCl, pH = 8.0, 137 mM NaCl, 2.7 mM KCl, 1 mM MgCl_2_, 1 mg/mL BSA). 15 µL of HDAC1 (0.4 µg/well) or HDAC6 (0.5 µg/well) were incubated at 37 °C for 30 minutes with 10 µL of inhibitor diluted in 5% DMSO and 25 µL of fluorogenic substrate (0.05 µL/well of 5 mM solution). Reactions were stopped with the developer and trichostatin A, diluted in buffer without BSA, and the plate was incubated at 30 °C for 45 min, followed by measuring the fluorescence (Ex. 360 nm, Em. 450 nm, Fluoroskan, Thermo Scientific).

Recombinant human protein kinase GST-CK2 was produced in bacteria and purified as described elsewhere. The phosphorylation reactions were conducted at 37 °C for 5 min in 50 uL samples each containing 1 pmol of human recombinant CK2α, 40 mM of peptide substrate (RRRADDSDDDDD), and an appropriate concentration of the tested compounds (0.1–200 µM). The reaction buffer contained 20 µM [γ-32P] ATP (specific radioactivity 300–1000 cpm/pmol), 15 mM Mg^2+^, 20 mM Tris–HCl pH 7.5, and 6 mM 2-mercaptoethanol. 10 µL of the assay mixture was spotted onto a square (1 cm/1 cm) of Whatman P81 paper and allowed to dry. Each square was immersed in cold 0.5% phosphoric acid, and washed three times during 10 min, allowed to dry and transferred into a vessel containing Opti-Phase liquid (scintillation solution) and radioactivity was quantified using a scintillation counter (MicroBeta, Perkin Elmer) by Cerenkov counting. All experiments were performed in triplicate. IC_50_ values were calculated using GraphPad Prism (version 4.0) software.

### 3.4. Cytotoxicity Studies

Human HCT-116 colon carcinoma cells, breast adenocarcinoma cells of MCF-7 line, Jurkat T-leukemia cells, leukemia cells of HL-60 cell line and its drug-resistant sublines HL-60/adr (overexpression of MRP-1) and HL-60/vinc (overexpression of P-glycoprotein) were kindly provided by Prof. Walter Berger, Institute of Cancer Research, Vienna Medical University. Human embryonal kidney cells of HEK293 line were obtained from cell culture collection of R.E. Kavetsky Institute of Experimental Pathology, Oncology and Radiobiology, National Academy of Sciences of Ukraine (Kyiv, Ukraine).

Cells were cultured in RPMI-1640 medium (Sigma-Aldrich, St. Louis, USA) supplemented with 10% fetal calf serum (Sigma-Aldrich, St. Louis, USA), 50 µg/mL streptomycin (Sigma-Aldrich, St. Louis, USA), and 50 units/mL penicillin (Sigma-Aldrich, St. Louis, USA) in 5% CO_2_ humidified atmosphere at 37 °C. Cells were seeded (5 × 10^5^/mL for leukemia cells and 1 × 10^5^/mL for carcinoma cells) into 24-well tissue culture plates (Greiner Bio-one, Frickenhausen, Germany) and were permitted to adhere and grow for 24 h before the experiment. Short-term (24 h) cytotoxic effect of antitumor drugs was studied under the Evolution 300 Trino microscope (Delta Optical, Nowe Osiny, Poland) after cell staining with trypan blue dye (0.1%).

Stock solutions (2 mM) of each CK2-HDAC inhibitor in DMSO (99.5% pure, Sigma, USA) were prepared, and additionally dissolved in serum-free culture medium (RPMI for leukemia cells and DMEM for carcinoma cells) prior to addition to cell culture. Final concentration of DMSO in cell culture was 0.5% or less. Cytotoxicity studies revealed no statistically significant toxicity of 0.5% DMSO solution on all cell lines, used in these studies.

### 3.5. Apoptosis Analysis

For cell death analyses, cells were stained with FITC-conjugated annexin V and propidium iodide (PI) using an apoptosis detection kit (BD Biosciences, San Jose, CA), according to the manufacturer’s instructions. In particular, 24 h after the addition of various concentration of CK2-HDAC inhibitors, Jurkat T-leukemia cells were centrifuged at 2,000 rpm, washed twice with 1x PBS, and incubated for 15 minutes in Annexin V binding buffer (BD Pharmingen, USA) containing 1/50 volume of FITC-conjugated Annexin V solution and PI (50 µg/mL). Then samples were diluted two times by appropriate volume of Annexin V binding buffer (BD Pharmingen, USA) and immediately measured on FL1/FL2 (FITC-PI) channels of FACScan flow cytometer (Becton Dickinson, USA). Analysis of the obtained results was carried out using the Cytomation Summit Software v3.1 (Cytomation Inc., USA).

### 3.6. Cell Cycle Analysis

Cell cycle of the analyzed cells was assessed according to protocol described by Walker et al. [97]. After drug treatment, 2 × 10^6^ cells were collected, pelleted by spinning at 1,000 rpm, 4 °C for five minutes, resuspended in 1 mL of cold PBS and fixed by adding drop by drop 4 mL of −20 °C absolute ethanol. On the next day, fixed cells were centrifuged again and cell pellets were resuspended in 1 mL of PBS. Then 100 µL of 200 µg/mL DNase-free, RNase A (Invitrogen, USA) was added to cell suspension and incubated at 37 °C for 30 minutes. After this 100 µL of 1 mg/mL propidium iodide was added to samples, which were incubated at room temperature for 5–10 min and then analyzed on FACScan flow cytometer (Becton Dickinson, USA). Cell cycle analysis was carried out using the Cytomation Summit Software v3.1 (Cytomation Inc., USA).

### 3.7. Studies of the Functional Status of Mitochondria

Breakdown of ΔΨm mitochondrial membrane potential was determined by FACS analysis using 5,5′,6,6′-tetrachloro-1,1′,3,3′-tetraethylbenzimidazolylcarbocyanine iodide (JC-1). For this purpose, the Mitochondrial Membrane Potential Detection Kit (Stratagene, La Jolla, CA, USA) was used as described in the manufacturer’s instruction. Briefly, 10^6^ Jurkat cells were treated for 1, 3, 6, 12 and 24 h with the tested drugs. After PBS washing, cells were incubated for 10 minutes in freshly prepared JC-1 solution (10 mg/mL in culture medium) at 37 °C. Spare dye was removed by PBS washing and cell-associated fluorescence was measured on FL1/FL2 (FITC-PI) channels of FACScan flow cytometer (Becton Dickinson, USA). Analysis of the obtained results was carried out using the Cytomation Summit Software v3.1 (Cytomation Inc., USA).

### 3.8. DCFDA and DHE Assays

Cellular ROS contents were measured by incubating control or drug-treated cells with fluorescent dyes dihydrodichlorofluorescein diacetate (DCFDA, detecting mainly H_2_O_2_) and dihydroethidium (DHE, O_2_^●−^-specific) in concentrations of 10 μM at 37 °C for 30 minutes. After incubation with the dyes, cells were washed with PBS and immediately analyzed at FL1 (DCFDA) or FL2 channel (DHE) of FACSCalibur flow cytometer (BD Biosciences, San Jose, CA).

### 3.9. Statistical Analyses

All experiments were performed in triplicate and repeated three times. Statistical analysis of data was conducted in GraphPad Prism 7.0 Software (GraphPad Software, Inc.) using Student’s t test. Statistical significance was set at P ≤ 0.05. Evaluation of LC_50_ values of each compound was done by built-in functions of GraphPad Prism (non-linear regression, curve fit, [Inhibitor] vs. normalized response - Variable slope).

## 4. Conclusions

In this study we demonstrate that a multitarget approach can provide interesting anticancer lead compounds. In silico studies showed plausible predicted binding modes for all the designed compounds in spite of their different dynamic behaviors. The best result for a dual CK2/HDAC1 inhibitor in the enzymatic assays was found for **11b** (CK2 IC_50_ = 1.46 µM; HDAC1 IC_50_ = 3.67 µM), a DMAT derivative with a five-carbon atom chain connecting the hydroxamate moiety. Furthermore, this compound was found to be even more active against HDAC6 (IC_50_ = 0.86 µM).

This compound showed a promising in vitro antiproliferative activity, with LC_50_ in the order of 4–23 µM in several tumor cell lines. Studies on the mechanism of cell death induction by **11b** showed apoptotic activity and G_1_/S growth arrest in Jurkat T-leukemia cells. This compound was also able to circumvent multidrug resistance of human leukemia cells, caused by overexpression of MRP-1 and P-glycoprotein, and this phenomenon was accompanied by an increase in superoxide production both in sensitive and MDR cell lines.

Compound **11d**, showing lower enzymatic activity against the three protein targets (CK2 IC_50_ = 5.89 µM; HDAC1 IC_50_ = 13.7 µM; HDAC6 IC_50_ = 8.98 µM), unexpectedly provided the best cellular activity with LC_50_ in 3–8 µM range. This difference in cellular behavior may be attributed to the increase in lipophilicity brought about by the longer seven-membered chain of **11d** compared to the five carbon linker of **11b**. Moreover, this inhibitor presented the highest cytotoxic activity, proapoptotic capability, and the best mitochondria-targeting and MDR circumventing properties, thus being the most promising drug candidate for further *in vivo* studies.

Altogether, this work further demonstrates that our multitarget HDAC/CK2 approach is a valid strategy for drug discovery in the field of anticancer drug development.

## Figures and Tables

**Table 1:** Inhibitory activity (IC_50_ (µM)) on HDAC1, HDAC6 and CK2.

Compound	HDAC1	HDAC6	CK2
**7a**	1.43	-	85.1
**7b**	2.25	2.41	50.1
**7c**	13.3	30.7	12.5
**7d**	10.2	-	53.8
**11a**	62.0	-	34.2
**11b**	1.46	0.66	3.67
**11c**	1.77	1.13	16.6
**11d**	13.7	8.98	5.89
**15a**	8.50	11.0	15.0
**15b**	4.22	2.70	136
**19**	9.45	20.6	7.15

**Table 2:** LC_50_ values of studied CK2-HDAC dual inhibitors towards human tumor and pseudonormal cell lines in vitro.

Comp	LC_50_ Values of Compounds for Cell Line, µM (M ± SD)						
	Jurkat	MCF-7	HCT-116	HEK293	HL-60	HL-60/adr	HL-60/vinc
**11b**	10.15 ± 1.43	15.66 ± 2.54	4.22 ± 0.46	23.33 ± 4.38	7.67 ± 1.17	16.23 ± 1.68	16.79 ± 3.34
**11c**	9.47 ± 1.10	9.97 ± 0.99	1.90 ± 0.24	13.61 ± 0.80	6.59 ± 0.96	10.63 ± 1.53	12.06 ± 3.27
**11d**	5.30 ± 0.75	9.02 ± 0.90	3.10 ± 0.37	8.41 ± 1.26	4.69 ± 0.54	8.4 ± 1.21	2.32 ± 1.06
**7c**	16.54 ± 2.33	27.75 ± 6.06	16.87 ± 3.90	42.71 ± 8.65	24.02 ± 8.66	18.58 ± 2.09	49.46 ± 9.47
**15a**	43.58 ± 16.73	13.66 ± 2.67	8.67 ± 1.02	33.73 ± 7.52	34.42 ± 7.89	32.37 ± 3.44	36.55 ± 4.74
**19**	24.86 ± 3.80	13.55 ± 0.78	11.3 ± 1.01	33.36 ± 11.76	46.49 ± 17.31	32.31 ± 5.32	21.73 ± 3.39

## Supplementary Materials

The following are available online, HPLC data; 13C NMR and 1H NMR of spectra of compounds **11a–d**, **7a–d**, **15a** and **19**. Figures S1–S7, Tables S1–S3 and Figure S8.

## Abbreviations

ATP	adenosine triphosphate
CK2	Protein kinase 2
BSA	Bovine Serum Albumin
CuAAC	Cu (I)-catalyzed alkyne azide cycloaddition
DCFDA	2′,7′ –dichlorofluorescin diacetate
DCM	dichloromethane
DMAT	2-dimethylamino-4,5,6,7-tetrabromo-1H-benzimidazole
DMF	dimethylformamide
DMSO	dimethyl sulfoxide
EDCI	1-ethyl-3-(3-dimethylaminopropyl)carbodiimide
EGFR	epidermal growth factor receptor
FACS	Fluorescence-activated cell sorting
FITC	Fluorescein isothiocyanate
HAT	histone acetyl transferase
HDAC	histone deacetylase
HER2	human epidermal growth factor receptor 2
HOBt	1-hydroxybenzotriazole
JAK2	Janus kinase 2
LC50	lethal concentration 50
LMD	molecular dynamics
MDR	multi-drug resistance
MTD	multitarget drugs
MW	microwave
NMM	*N*-Methylmorpholine
OTHP	2-tetrahydropyranyl
PI3K	osfoinositol 3-quinasa
RMSD	root-mean-square deviation
ROS	reactive oxygen species
RT	room temperature
SAR	structure activity relationship
SRM	surface recognition moiety
TBB	tetrabromobenzotriazole
TBI	tetrabromobenzimidazole
TBTA	Tris(benzyltriazolyl)methylamine
THF	Tetrahydrofuran
TsOH	*p*-Toluenesulfonic acid
ZBG	Zinc binding group
