# Supplementary material for: Multitarget Anticancer Agents Based on Histone Deacetylase and Protein Kinase CK2 Inhibitors
Source: Molecules. 2020 Mar 25;25(7):1497. doi: 10.3390/molecules25071497 (PMC7180456; doi:10.3390/molecules25071497)
Supplement: Supplementary file 1 [file molecules-25-01497-s001.pdf]

Supporting Information

Multi-target Anticancer Agents Based on Histone Deacetylase and Protein Kinase CK2 inhibitors

Regina Martínez [1], Bruno Di Geronimo [1], Miryam Pastor [1], José María Zapico [1], Claire Coderch [1], Rostyslav Panchuk [2], Nadia Skorokhyd [2], Maciej Maslyk [3], Ana Ramos [1,] and Beatriz de Pascual-Teresa [1,*]*

[1] Departamento de Química y Bioquímica, Facultad de Farmacia, Universidad San Pablo-CEU, CEU Universities, Urbanización Monteprincipe, 28925, Alcorcón, Madrid, Spain.
[2] Institute of Cell Biology, NAS of Ukraine, Drahomanov Str. 14/16, 79005, Lviv, Ukraine.
[3] Department of Molecular Biology, Faculty of Biotechnology and Environment Sciences, The John Paul II Catholic University of Lublin, 20-718, Lublin, Poland.

Table of Contents:

		Pages
1	HPLC data; ^{13}C NMR and ^{1}H NMR of spectra of compounds **11a-d**, **7a-d**, **15a** and **19.**	S2-S17
2	**Figure 1S**	S18-S19
3	**Figure 2S**	S20
3	**Figure 3S**	S21
4	**Figure 4S**	S22
5	**Figure 5S**	S23
6	**Figure 6S**	S24-S25
7	**Figure 7S**	S26-S27
8	**Table 1S**	S28
9	**Table 2S**	S29
10	**Table 3S**	S30
11	**Table 4S**	S32
12	**Figure 8S**	S32

N-Hydroxy-5-(4,5,6,7-tetrabromo-2-(dimethylamino)-1H-benzo[d]imidazol-1-yl)pentanamide (11a)

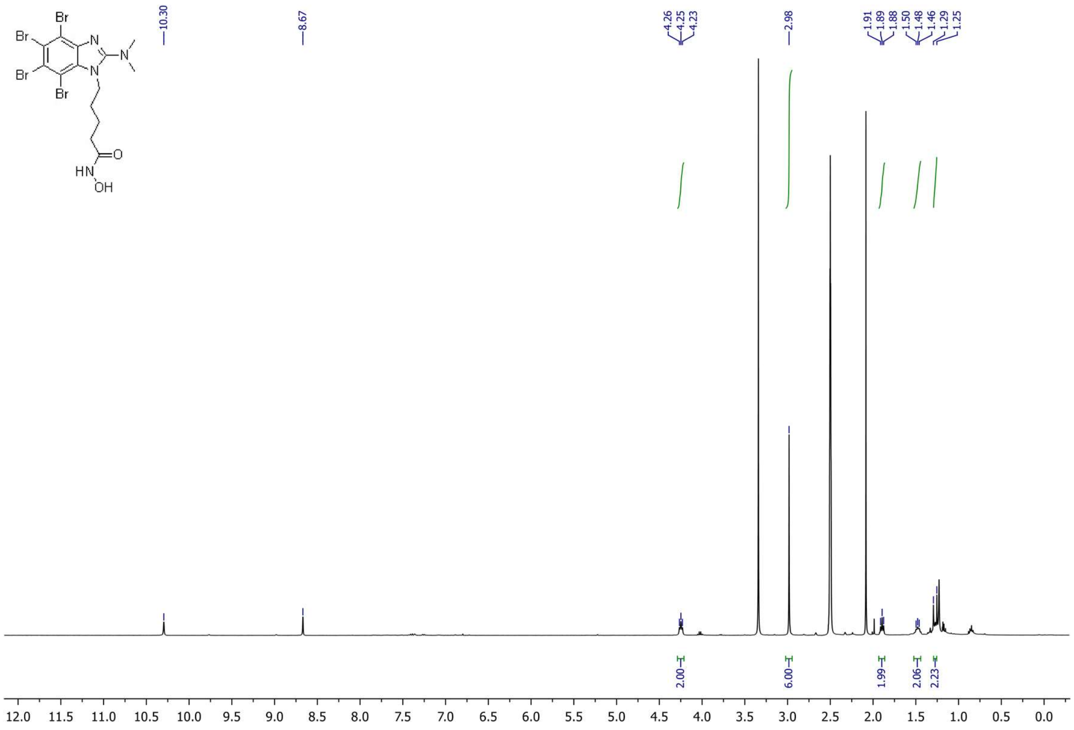

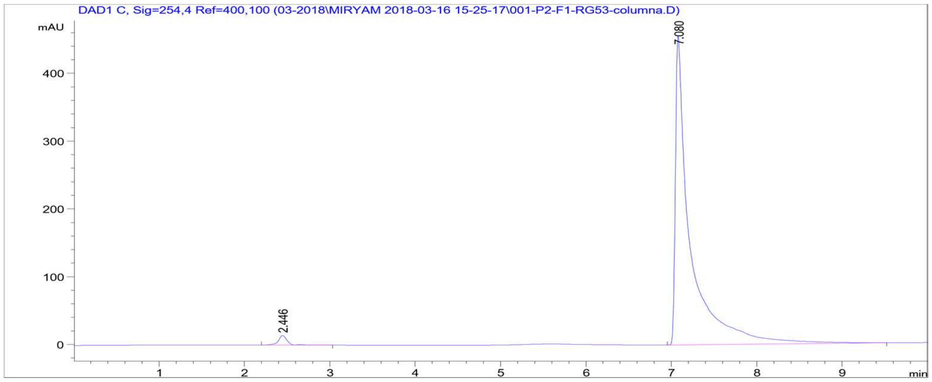

Signal 1: DAD1 C, Sig=254,4 Ref=400,100

Peak #	RetTime [min]	Type	Width [min]	Area [mAU*s]	Height [mAU]	Area %
1	2.446	BV R	0.1081	102.73285	14.24945	1.8137
2	7.080	BB	0.1603	5561.46387	456.00397	98.1863

Totals : 5664.19672 470.25342

N-Hydroxy-6-(4,5,6,7-tetrabromo-2-(dimethylamino)-1H-benzo[d]imidazol-1-yl)hexanamide (11b)

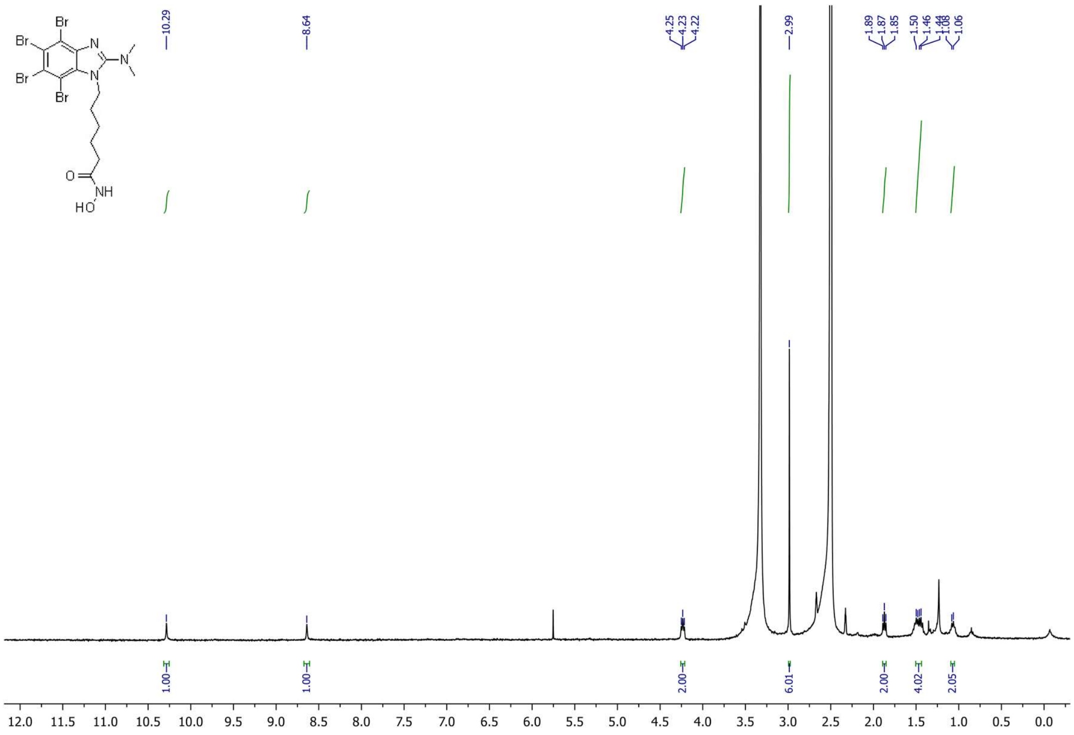

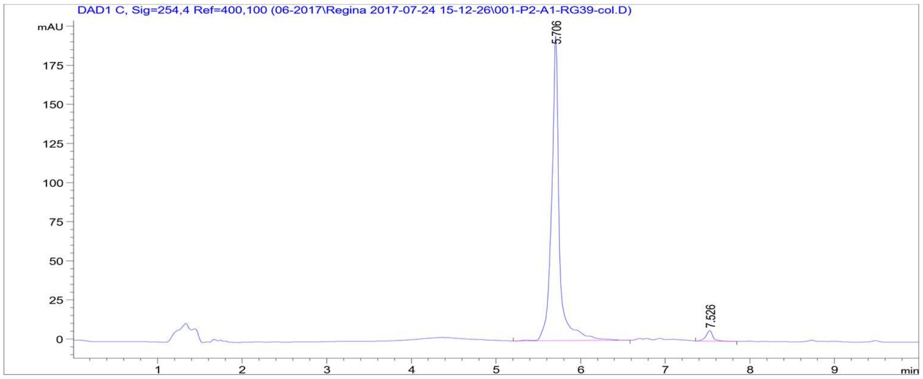

Signal 1: DAD1 C, Sig=254,4 Ref=400,100

Peak #	RetTime [min]	Type	Width [min]	Area [mAU*s]	Height [mAU]	Area %
1	5.706	BB	0.0911	1255.41211	195.07144	97.0298
2	7.526	BB	0.0840	38.43026	6.79341	2.9702
Totals :				1293.84237	201.86485	

N-Hydroxy-7-(4,5,6,7-tetrabromo-2-(dimethylamino)-1H-benzo[d]imidazol-1-yl)heptanamide (11c)

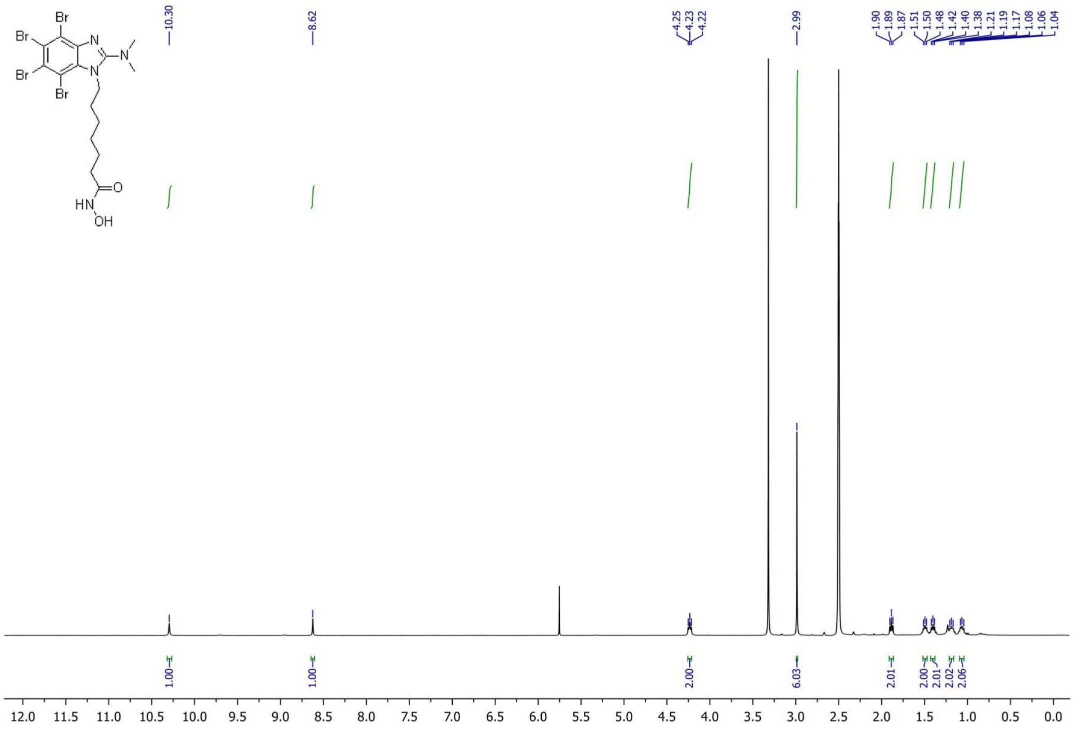

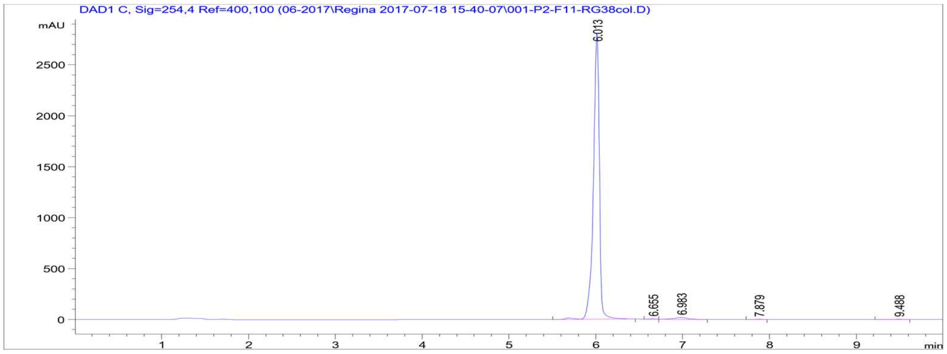

Signal 1: DAD1 C, Sig=254,4 Ref=400,100

Peak #	RetTime [min]	Type	Width [min]	Area [mAU*s]	Height [mAU]	Area %
1	6.013	VB R	0.0714	1.34746e4	2814.50049	98.0701
2	6.655	BV	0.0582	21.47060	5.66641	0.1563
3	6.983	VB	0.1598	206.18138	19.43099	1.5006
4	7.879	BB	0.0677	15.60688	3.52331	0.1136
5	9.488	BB	0.0879	21.90463	3.65501	0.1594

Totals : 1.37397e4 2846.77620

***N*-Hydroxy-8-(4,5,6,7-tetrabromo-2-(dimethylamino)-1*H*-benzo[*d*]imidazol-1-yl)octanamide (11d)**

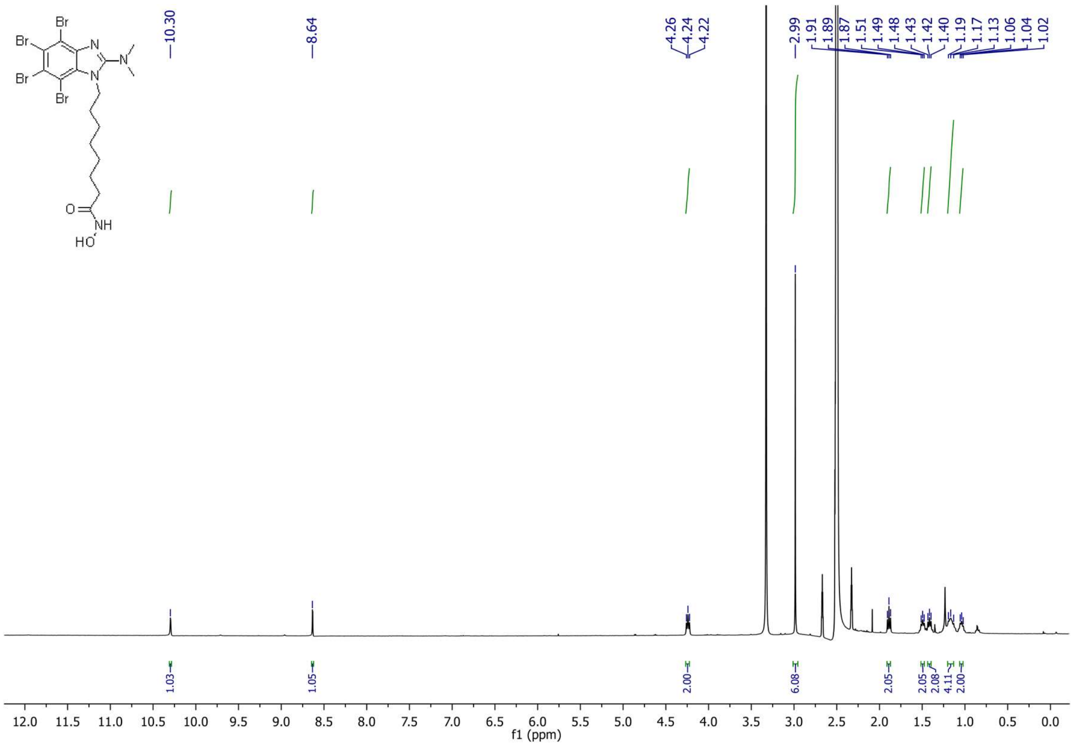

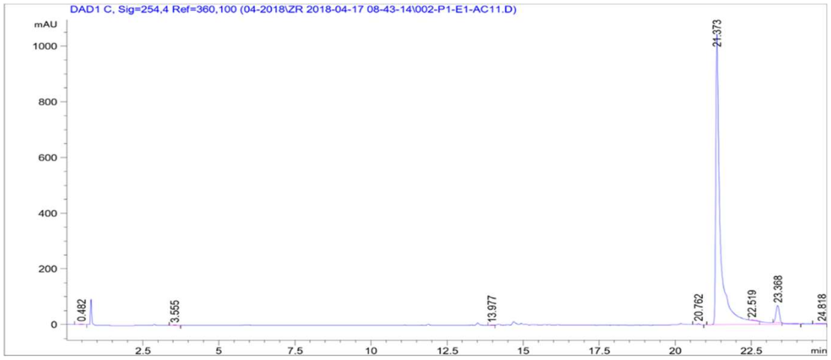

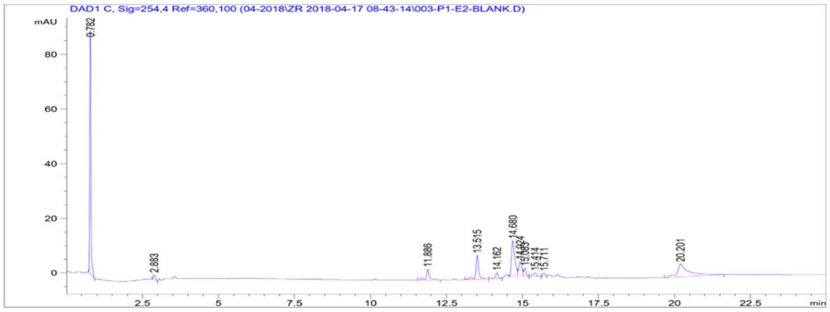

Signal 1: DAD1 C, Sig=254,4 Ref=360,100

Peak #	RetTime [min]	Type	Width [min]	Area [mAU*s]	Height [mAU]	Area %
1	0.482	BB	0.1579	19.23498	1.93644	0.1646
2	3.555	BB	0.0685	5.18632	1.15253	0.0444
3	13.977	BV	0.0945	6.41406	1.00500	0.0549
4	20.762	BB	0.0939	20.38050	3.30734	0.1744
5	21.373	BV R	0.1458	1.11087e4	1046.84460	95.0811
6	22.519	VV E	0.1938	26.92827	1.80767	0.2305
7	23.368	VB E	0.1185	473.02039	62.33468	4.0487
8	24.818	BBA	0.2264	23.52219	1.35572	0.2013
Totals :				1.16833e4	1119.74398	

***N*-Hydroxy-4-(4-((4,5,6,7-tetrabromo-2-(dimethylamino)-1*H*-benzo[*d*]imidazol-1-yl)methyl)-1*H*-1,2,3-triazol-1-yl)butanamide (7a)**

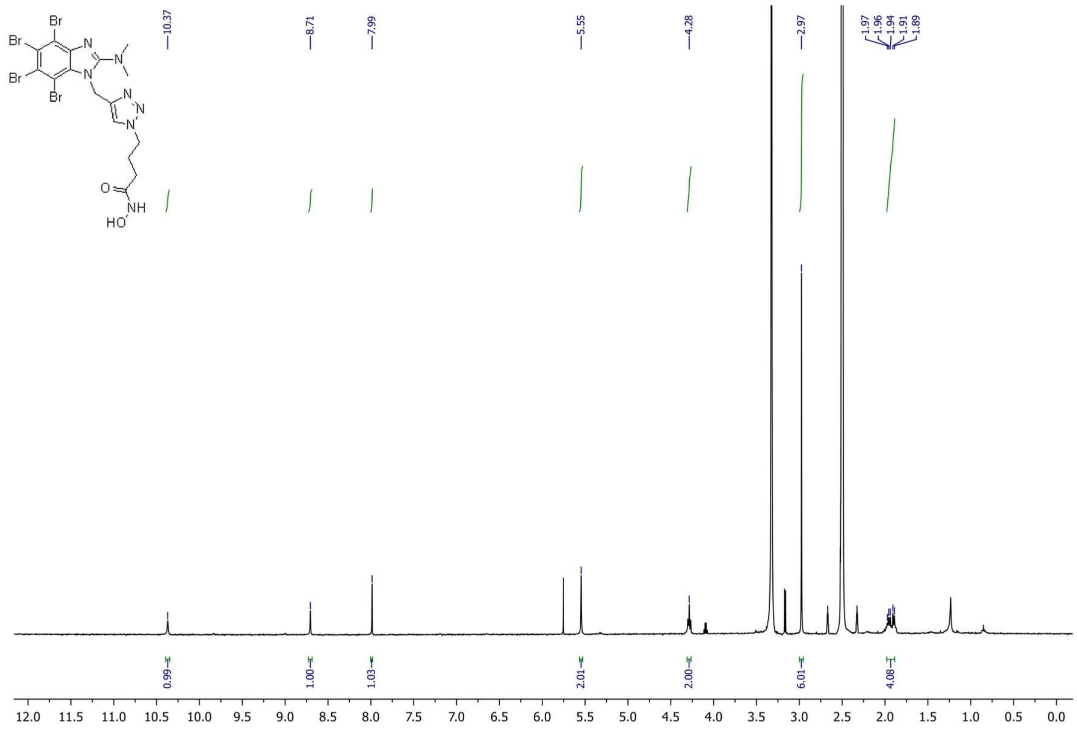

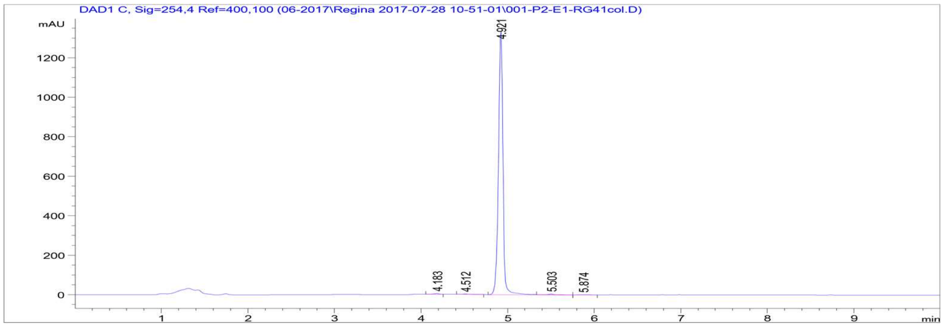

Signal 1: DAD1 C, Sig=254,4 Ref=400,100

Peak #	RetTime [min]	Type	Width [min]	Area [mAU*s]	Height [mAU]	Area %
1	4.183	BB	0.0585	12.17241	3.19194	0.2530
2	4.512	BB	0.0889	9.41010	1.46527	0.1956
3	4.921	BV R	0.0556	4761.73730	1336.29236	98.9677
4	5.503	VV E	0.0708	19.04000	3.91370	0.3957
5	5.874	VB	0.1027	9.04547	1.18934	0.1880

Totals : 4811.40529 1346.05262

***N*-Hydroxy-5-(4-((4,5,6,7-tetrabromo-2-(dimethylamino)-1*H*-benzo[*d*]imidazol-1-yl)methyl)-1*H*-1,2,3-triazol-1-yl)pentanamide (7b)**

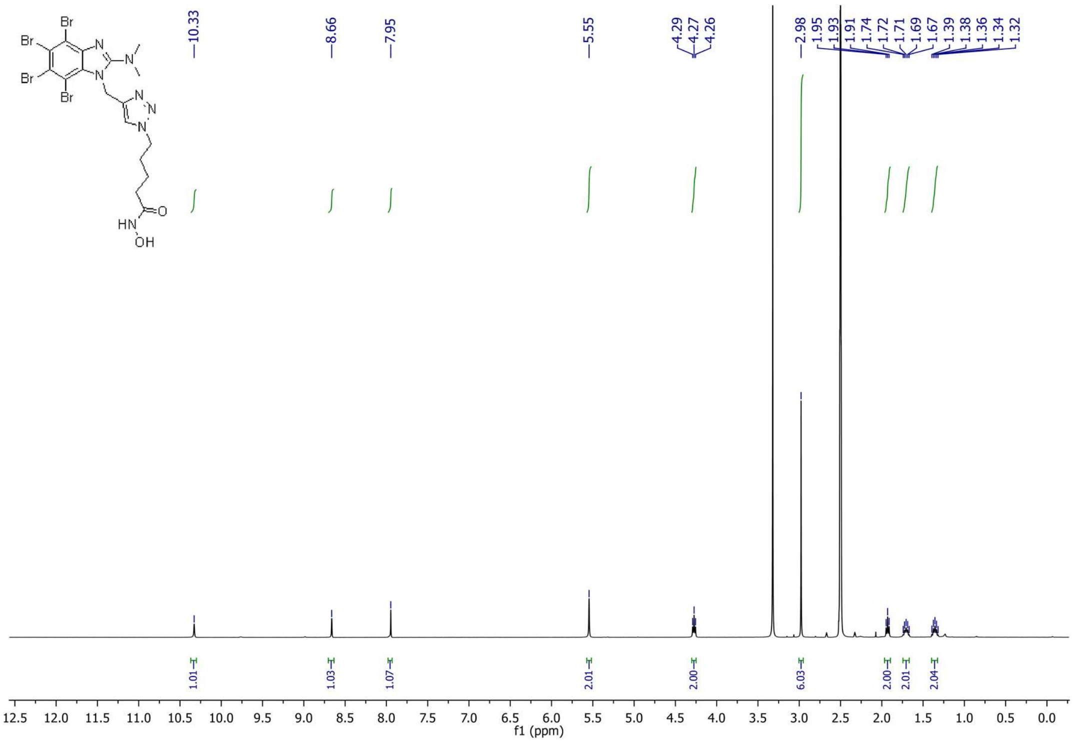

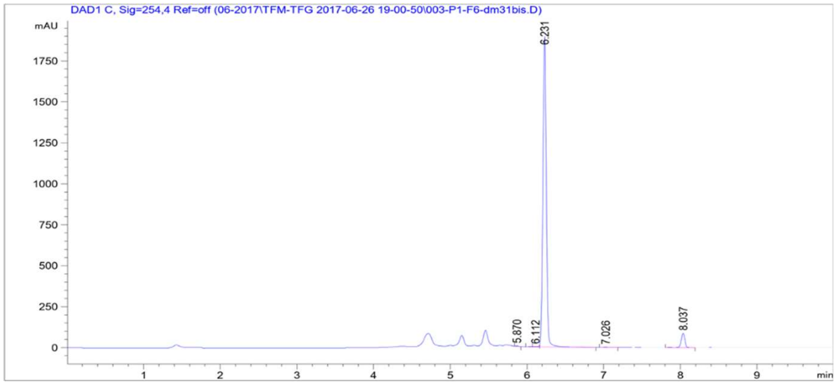

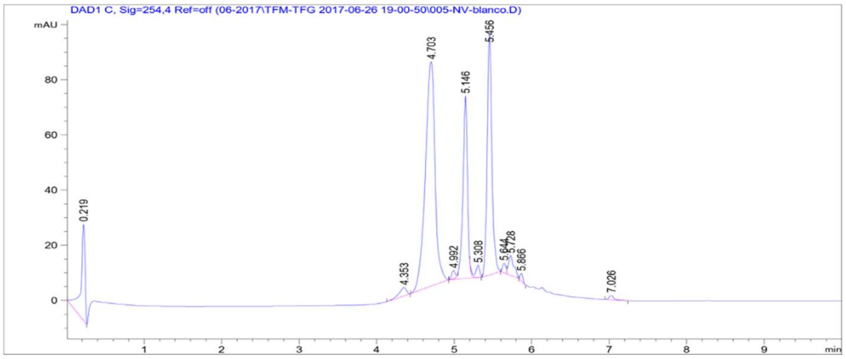

Signal 1: DAD1 C, Sig=254,4 Ref=off

Peak #	RetTime [min]	Type	Width [min]	Area [mAU*s]	Height [mAU]	Area %
1	5.870	BB	0.0412	7.60626	3.03399	0.1215
2	6.112	VV E	0.0573	10.10914	2.72607	0.1615
3	6.231	VB R	0.0506	5961.39844	1900.22595	95.2432
4	7.026	BB	0.0629	7.36980	1.75839	0.1177
5	8.037	VB R	0.0502	272.64691	87.76031	4.3560
Totals :				6259.13055	1995.50472	

N-Hydroxy-3-(4-(2-(4,5,6,7-tetrabromo-2-(dimethylamino)-1H-benzo[d]imidazol-1-yl)ethyl)-1H-1,2,3-triazol-1-yl)propanamide (7c)

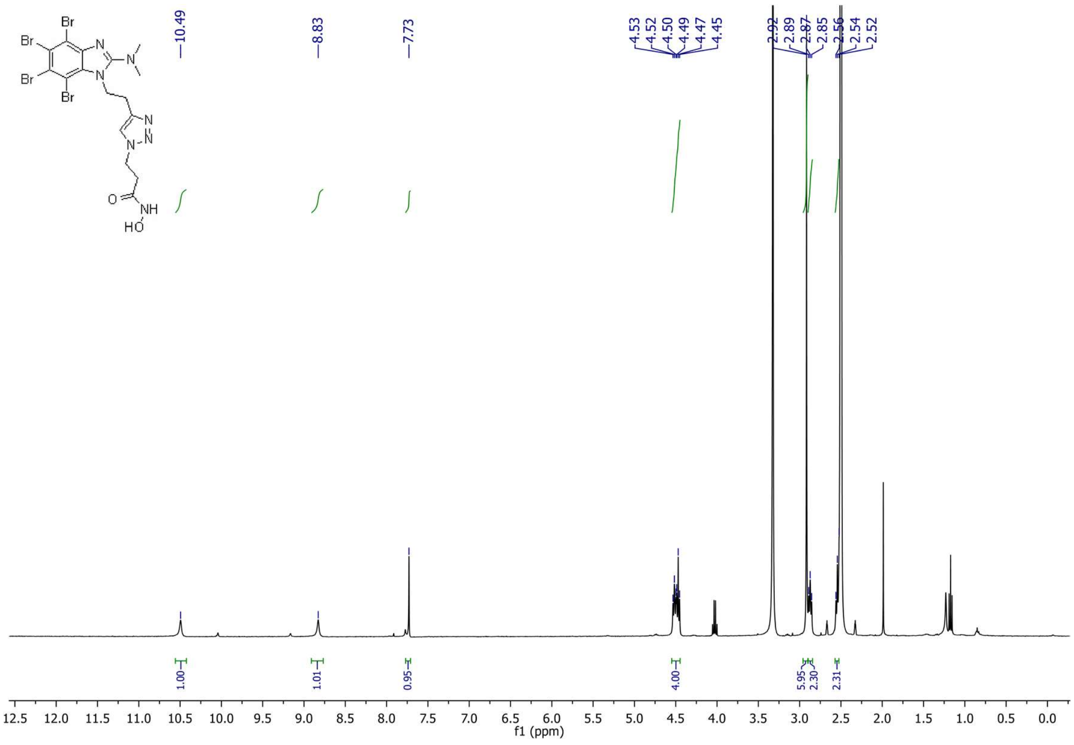

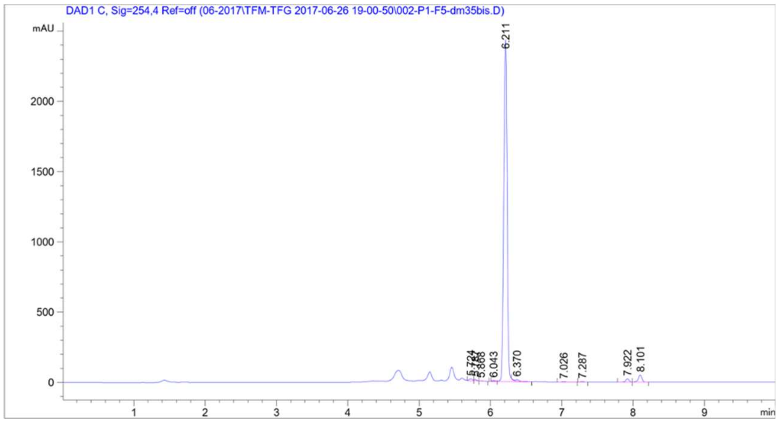

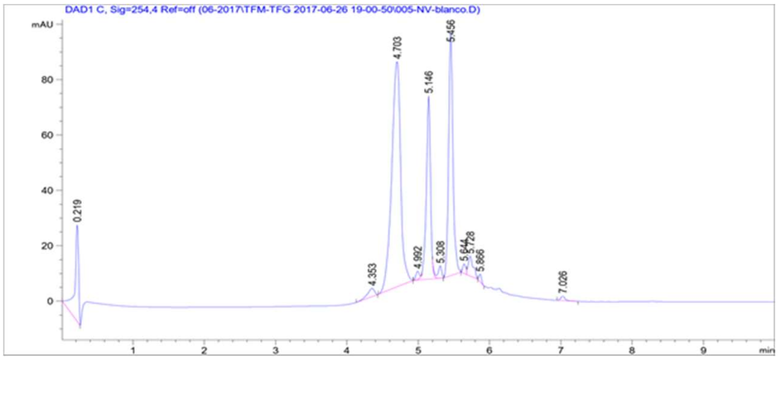

Signal 1: DAD1 C, Sig=254,4 Ref=off

Peak #	RetTime [min]	Type	Width [min]	Area [mAU*s]	Height [mAU]	Area %
1	5.724	VV	0.0561	50.68787	13.39521	0.6075
2	5.787	VB	0.0423	25.12651	9.06702	0.3011
3	5.868	BB	0.0421	5.56397	2.15571	0.0667
4	6.043	BV E	0.0435	10.05735	3.97738	0.1205
5	6.211	VV R	0.0518	7909.91650	2442.92969	94.7956
6	6.370	VB E	0.0670	55.43245	11.76695	0.6643
7	7.026	BB	0.0658	7.73054	1.73999	0.0926
8	7.287	BB	0.0487	15.26694	5.13314	0.1830
9	7.922	BV	0.0513	80.69362	23.95649	0.9671
10	8.101	VB	0.0539	183.70345	51.14900	2.2016

Totals : 8344.17919 2565.27057

***N*-Hydroxy-4-(4-(2-(4,5,6,7-tetrabromo-2-(dimethylamino)-1*H*-benzo[*d*]imidazol-1-yl)ethyl)-1*H*-1,2,3-triazol-1-yl)butanamide (7d)**

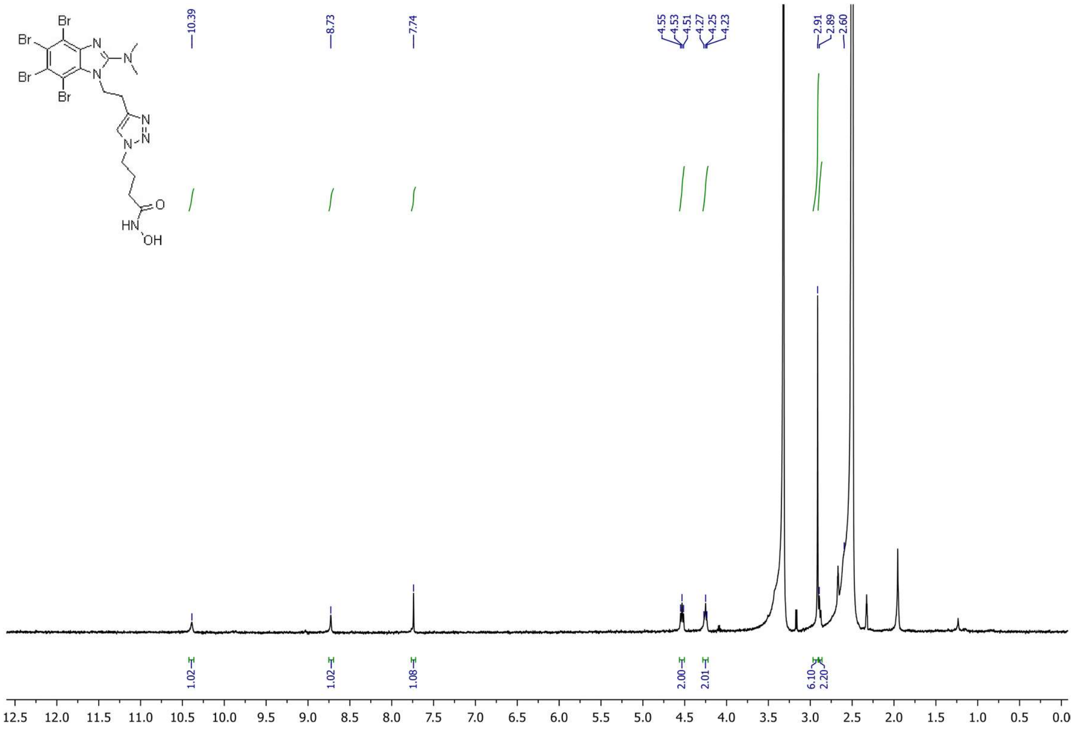

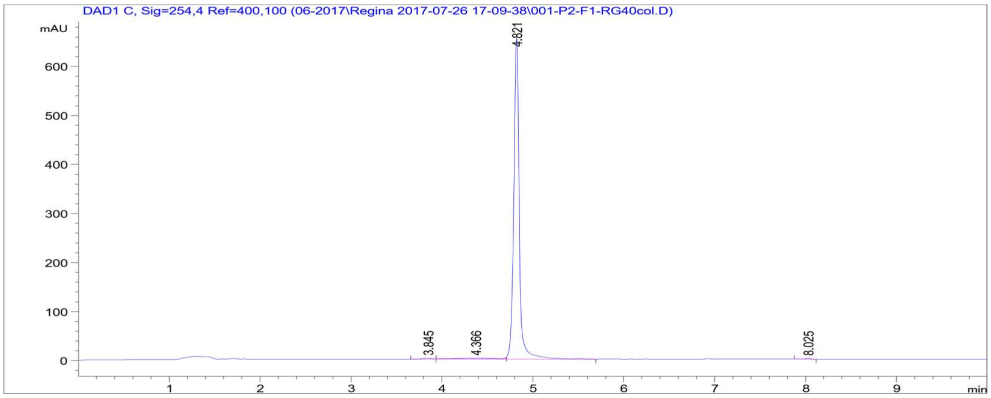

Signal 1: DAD1 C, Sig=254,4 Ref=400,100

Peak #	RetTime [min]	Type	Width [min]	Area [mAU*s]	Height [mAU]	Area %
1	3.845	BB	0.0823	10.92274	1.92117	0.3890
2	4.366	BV E	0.3453	48.39608	1.87415	1.7234
3	4.821	VV R	0.0628	2742.30640	655.64117	97.6563
4	8.025	BB	0.0624	6.49529	1.63441	0.2313

Totals : 2808.12051 661.07091

_N_-hydroxy-4-((perbromo-2_H_-benzo[_d_][1,2,3]triazol-2-yl)methyl)benzamide (15a)

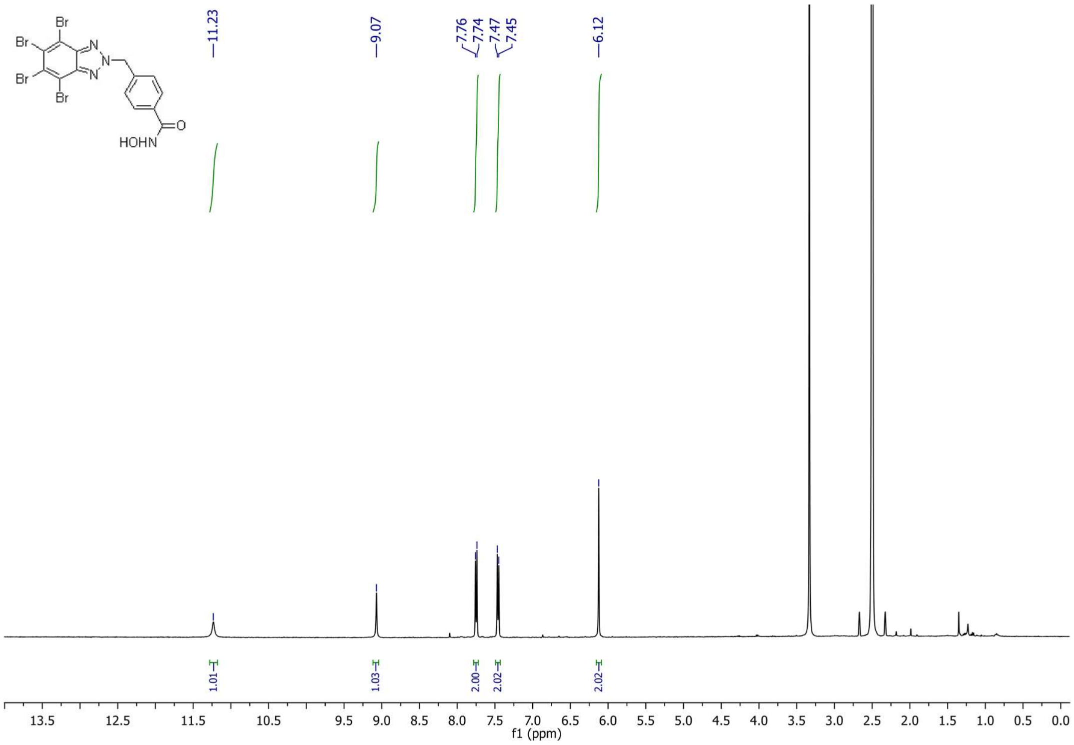

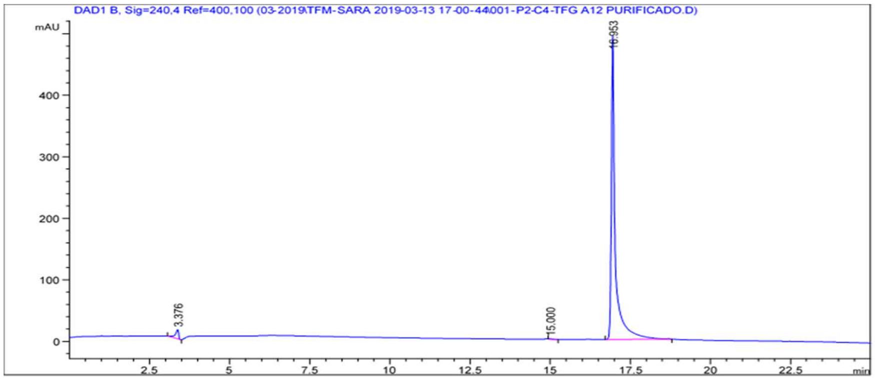

Signal 1: DAD1 B, Sig=240,4 Ref=400,100

Peak #	RetTime [min]	Type	Width [min]	Area [mAU*s]	Height [mAU]	Area %
1	3.376	BB	0.1028	108.81815	14.97678	2.5030
2	15.000	VB	0.0794	7.21641	1.37051	0.1660

Peak #	RetTime [min]	Type	Width [min]	Area [mAU*s]	Height [mAU]	Area %
3	16.953	BV R	0.1201	4231.44922	493.33005	97.3310

Totals : 4347.48378 509.67734

N-hydroxy-4-((perbromo-1H-benzo[d][1,2,3]triazol-1-yl)methyl)benzamide (15b)

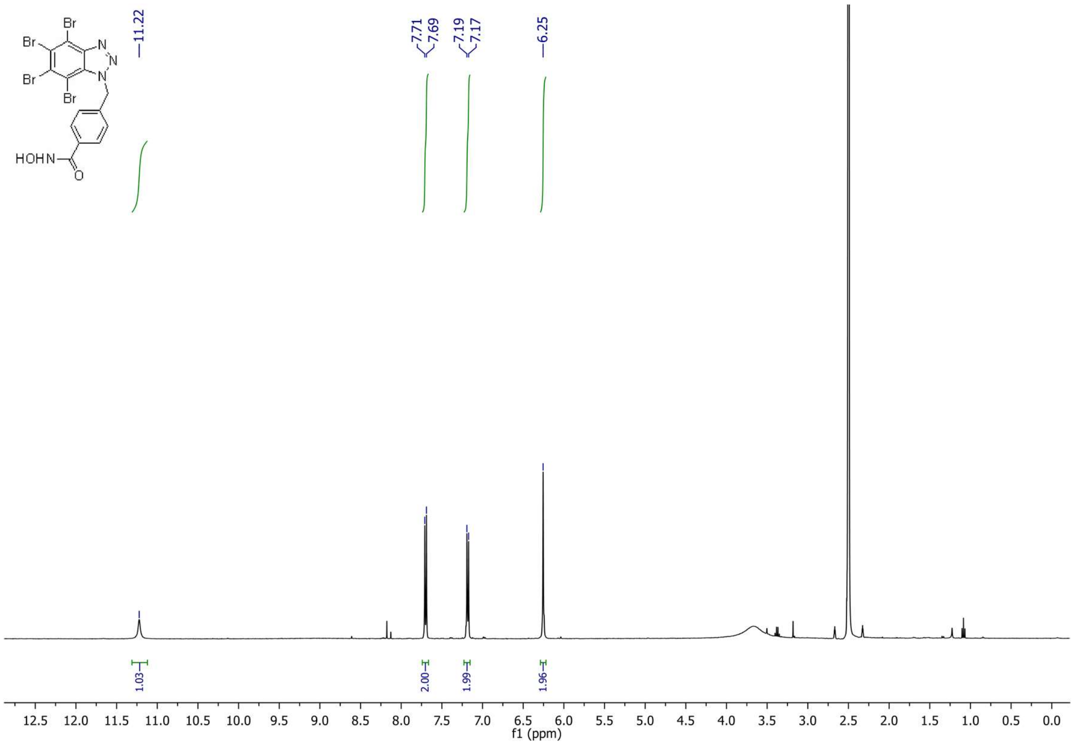

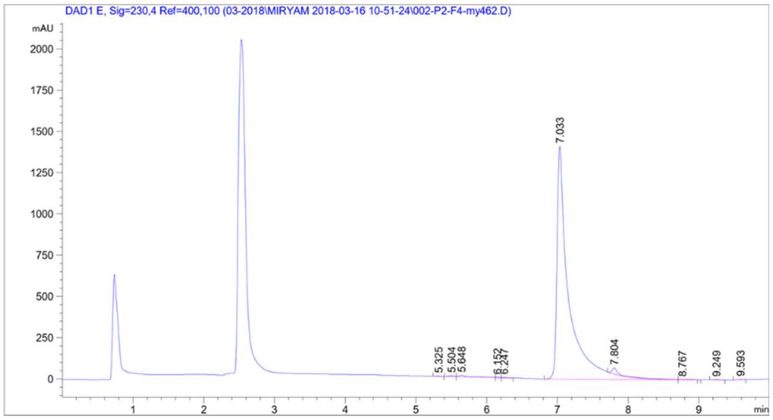

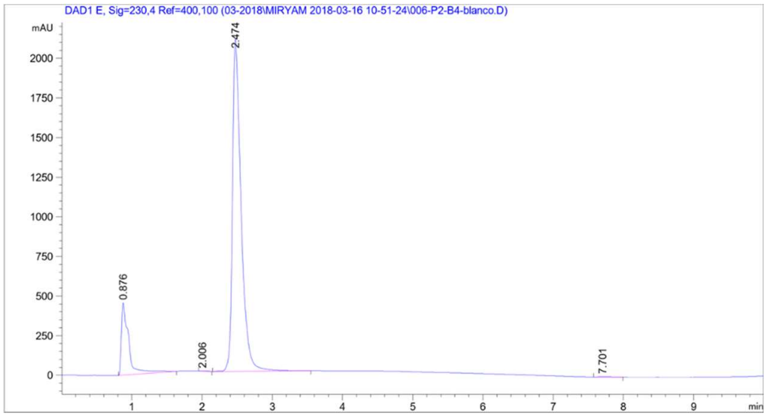

Signal 1: DAD1 E, Sig=230,4 Ref=400,100

Peak #	RetTime [min]	Type	Width [min]	Area [mAU*s]	Height [mAU]	Area %
1	5.325	BB	0.0810	9.72930	2.06216	0.0581
2	5.504	BV	0.0694	26.04988	5.69306	0.1555
3	5.648	VV	0.1317	85.87582	8.96607	0.5125
4	6.152	VV	0.0642	8.05488	1.87321	0.0481
5	6.247	VB	0.0618	10.84912	2.64667	0.0648
6	7.033	BV R	0.1547	1.62896e4	1410.75134	97.2238
7	7.804	VV E	0.1023	276.67804	37.41324	1.6513
8	8.767	VB E	0.1558	17.99244	1.59307	0.1074
9	9.249	BB	0.0814	16.90264	3.21241	0.1009
10	9.593	BB	0.0756	13.00847	2.72793	0.0776
Totals :				1.67548e4	1476.93917	

***N*-hydroxy-4-((4,5,6,7-tetrabromo-2-(dimethylamino)-1*H*-benzo[*d*]imidazol-1-yl)methyl)benzamide (19)**

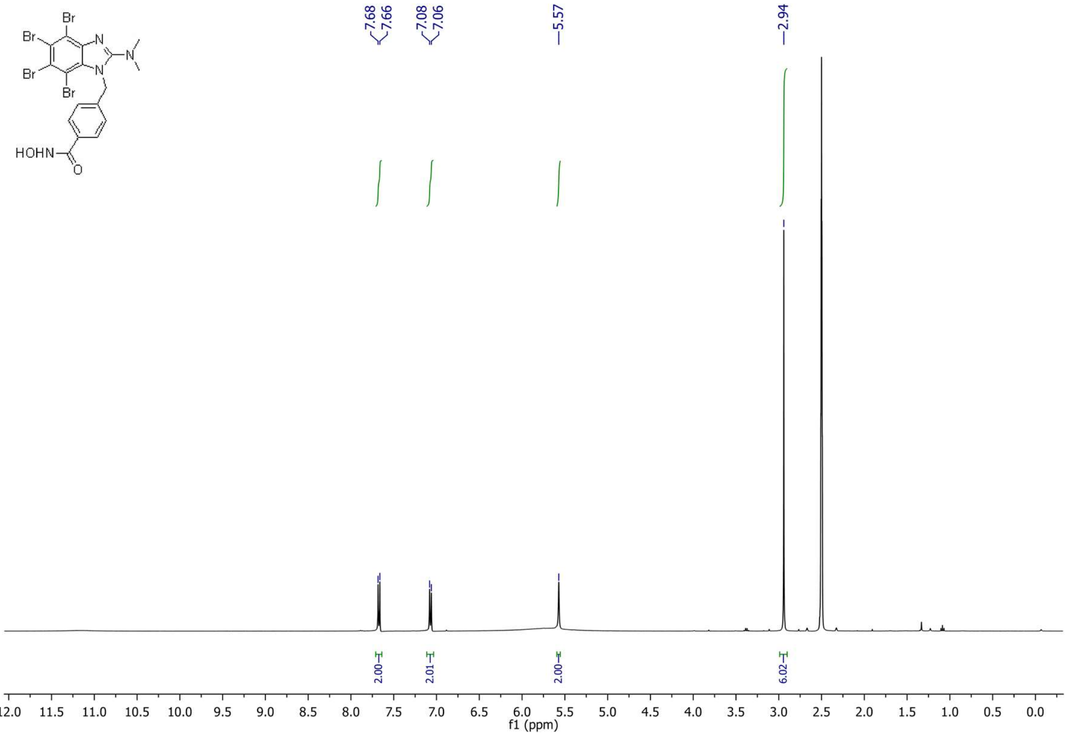

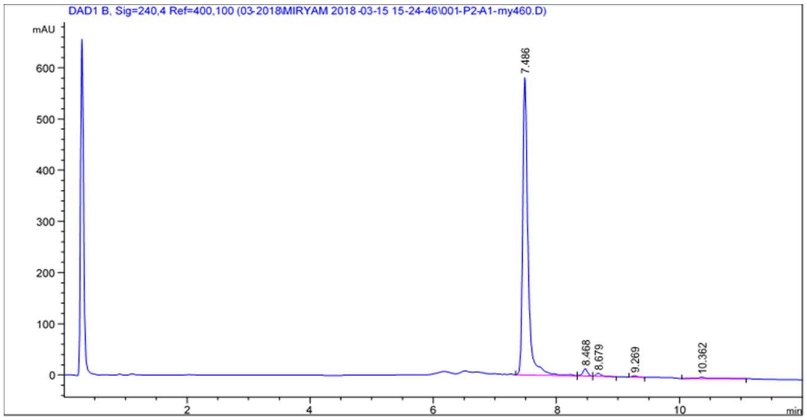

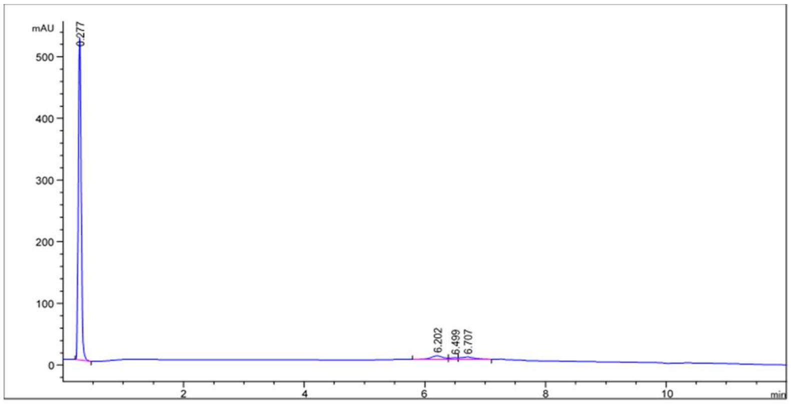

Signal 1: DAD1 B, Sig=240,4 Ref=400,100

Peak #	RetTime [min]	Type	Width [min]	Area [mAU*s]	Height [mAU]	Area %
1	7.486	BV R	0.0878	3493.92505	581.92426	94.9975
2	8.468	VB	0.0782	75.26643	14.11689	2.0464
3	8.679	BB	0.0847	34.41696	6.02324	0.9358
4	9.269	BB	0.0808	14.32347	2.74979	0.3894
5	10.362	BB	0.2474	59.98135	3.07677	1.6309
Totals :				3677.91326	607.89094	

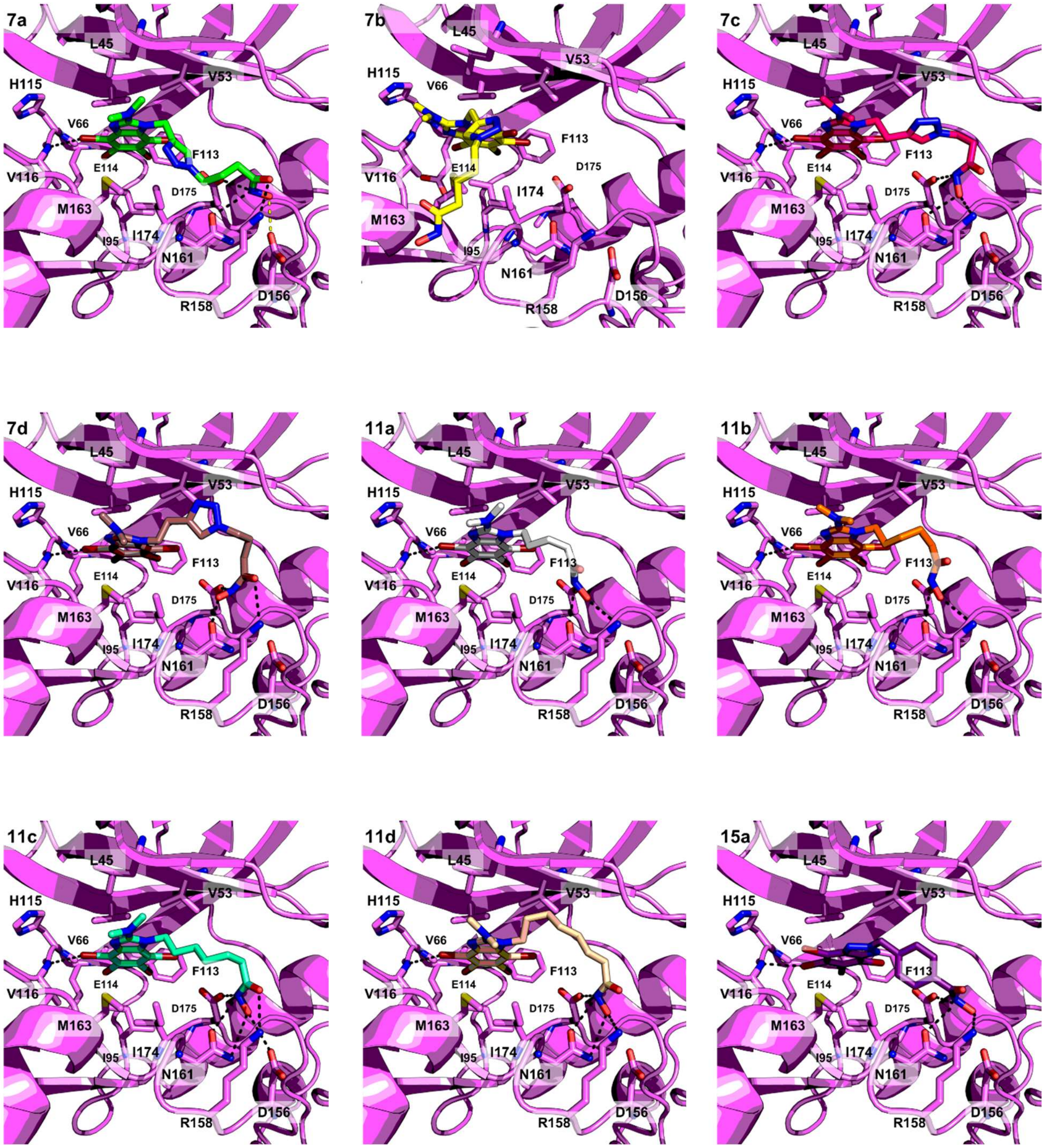
7a
L45
V53
H115
V66
F113
E114
V116
D175
M163
I95
I174
N161
R158
D156
7b
I174
D175
7c
7d
11a
11b
11c
11d
15a

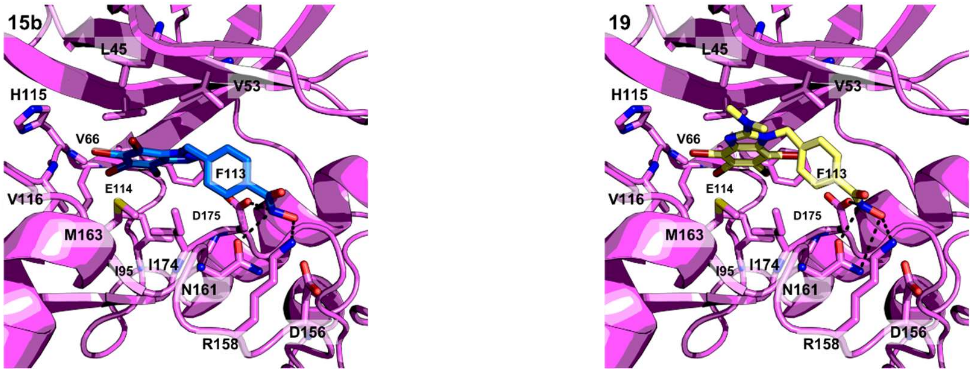

Figure 1S: PyMOL stick and cartoon representation of the best docking poses obtained for the set of compounds in CK2. For the sake of clarity, only heavy atoms are shown.

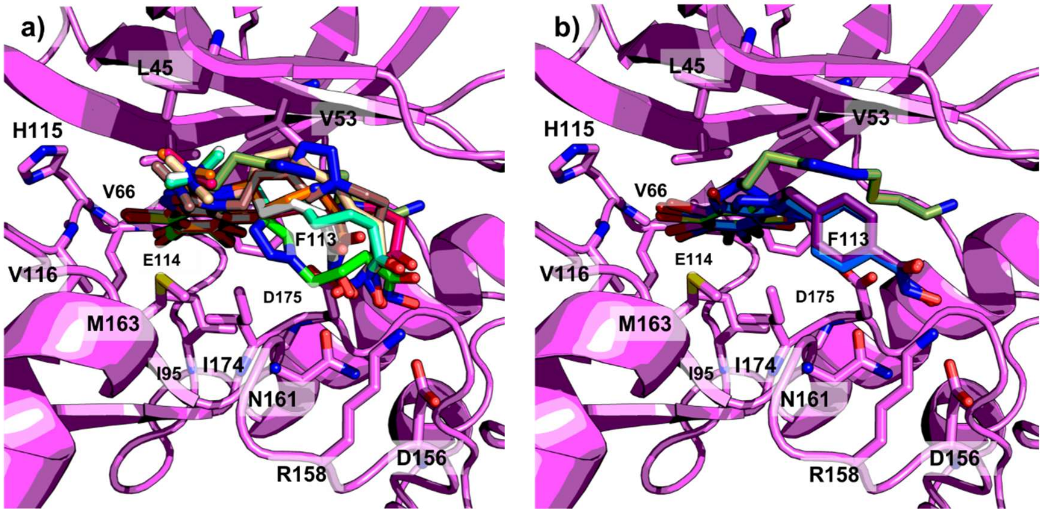

Figure 2S: PyMOL stick and cartoon representation of the best docking poses compared with the binding of the reference compound **JRJ** (colored in olive green) within CK2. **a)** binding of compounds **11a-d, 7a, 7d** and **19** that achieve a perfect superimposition of the TBI moiety with that of **JRJ**. **b)** binding of compounds **15a-b** that present a different orientation of the TBB moiety compared to reference compound **JRJ**.

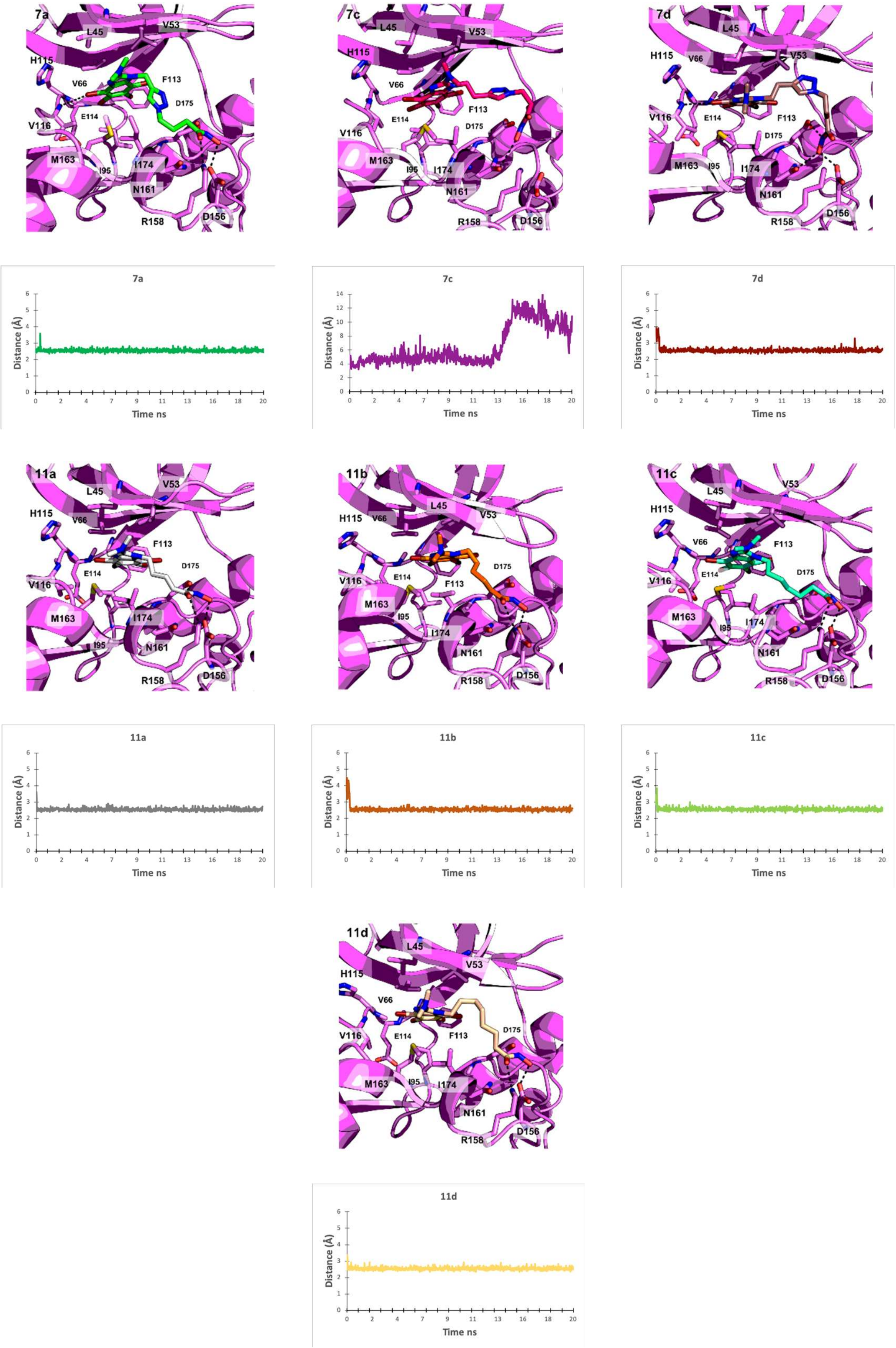

Figure 3S. PyMOL sick and cartoon representation of the complexes of the most populated conformers of compounds **7a**, **7c, 7d** and **11a-d** with CK2. Graphical representations of the evolution during the entire simulation time (ns) of the hydrogen bond interaction established between the hydroxamic acid and the side chain of Asp156.

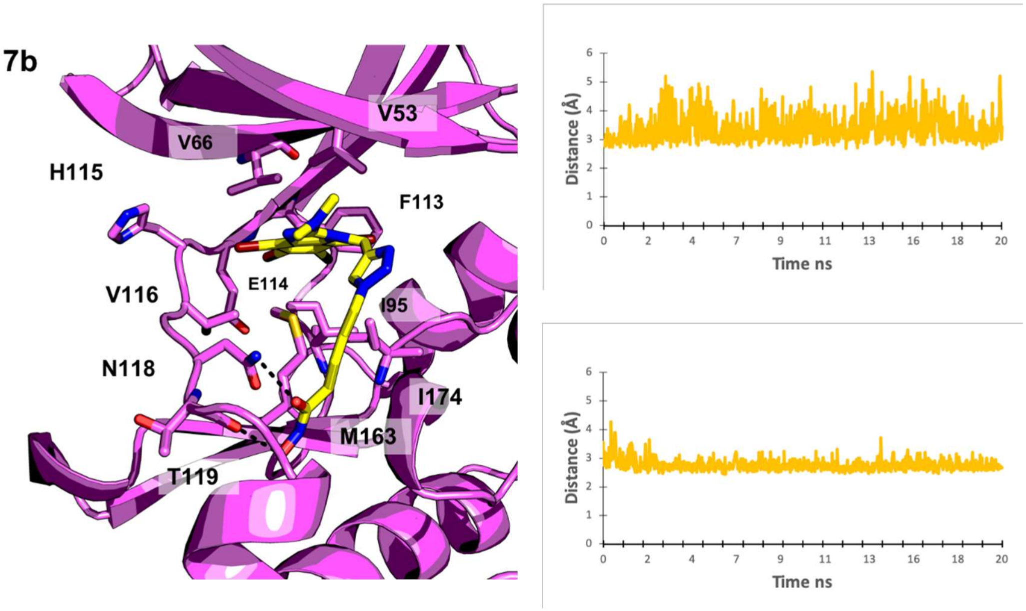

Figure 4S: PyMOL sick and cartoon representation of the complex of most the populated conformer of compound **7b** within CK2. Graphical representations of the evolution during the entire simulation time (ns) of the hydrogen bond interaction established between the hydroxamic acid and the side chain and backbone of Asn118 (top) and Thr119 (bottom), respectively.

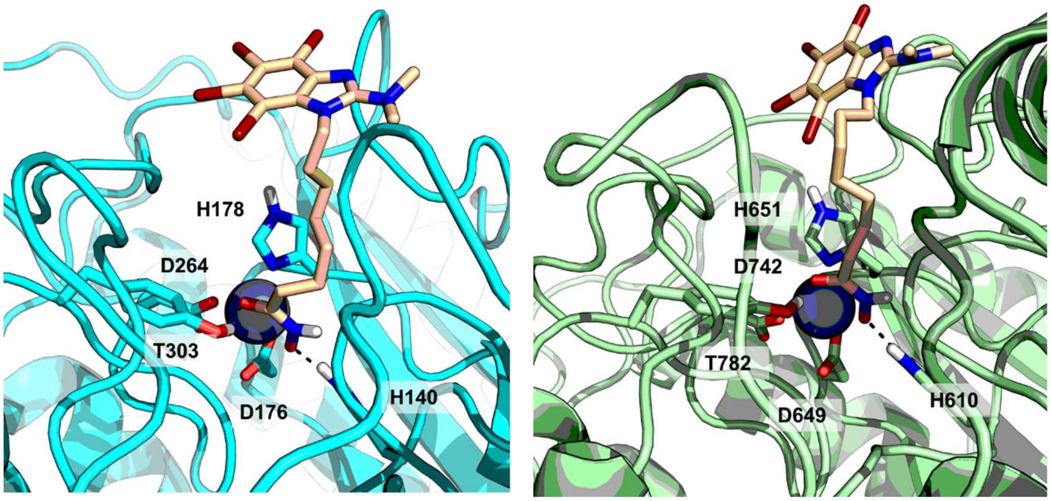

Figure 5S: PyMOL stick and cartoon representation of the bidentate chelation and hydrogen bond stabilization to the catalytic site of HDAC1 (left) and HDAC6 (right) of the hydroxamate of compound **11d** selected as representation of the binding of all of the compounds studied.

7a

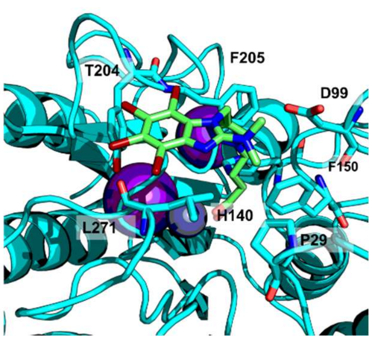

7b

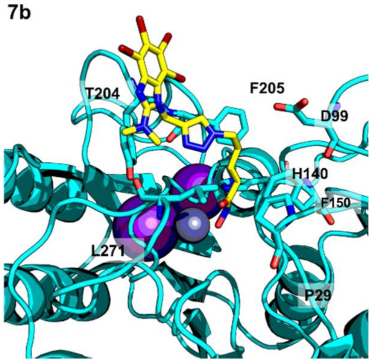

7c

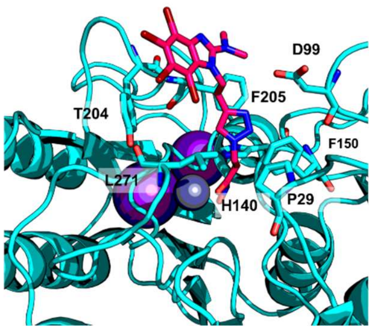

7d

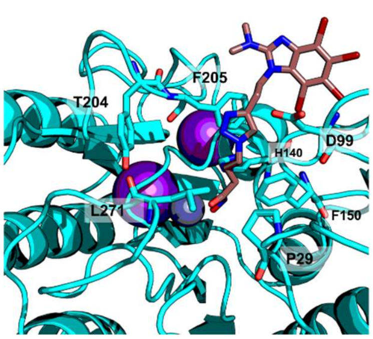

11a

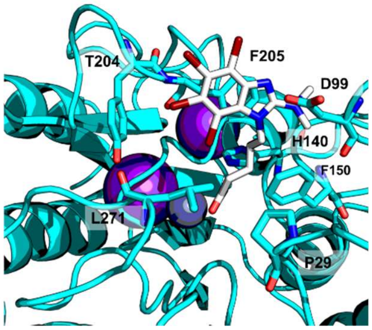

11b

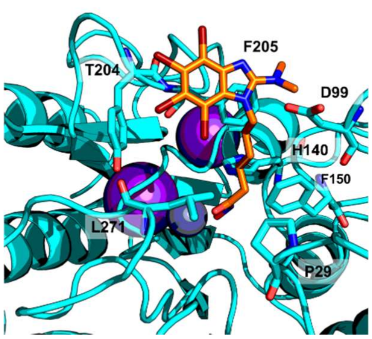

11c

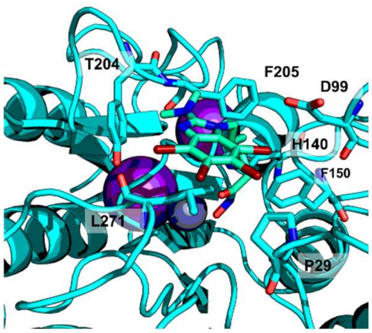

11d

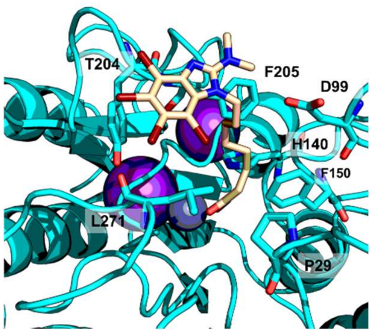

15a

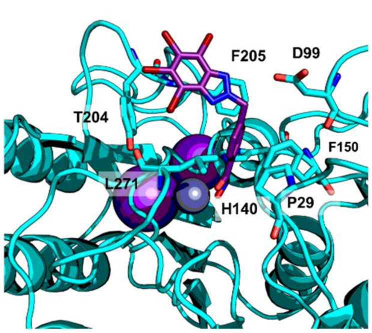

15b **19**

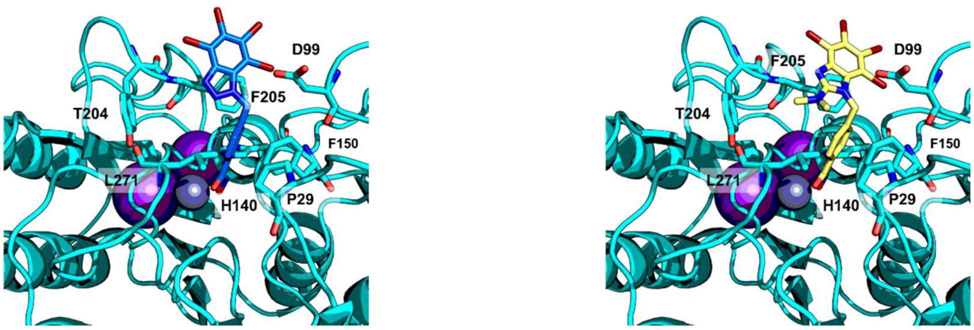

Figure 6S: PyMOL stick and cartoon representation of the best docking poses obtained for the set of compounds in HDAC1. For the sake of clarity only heavy atoms are shown.

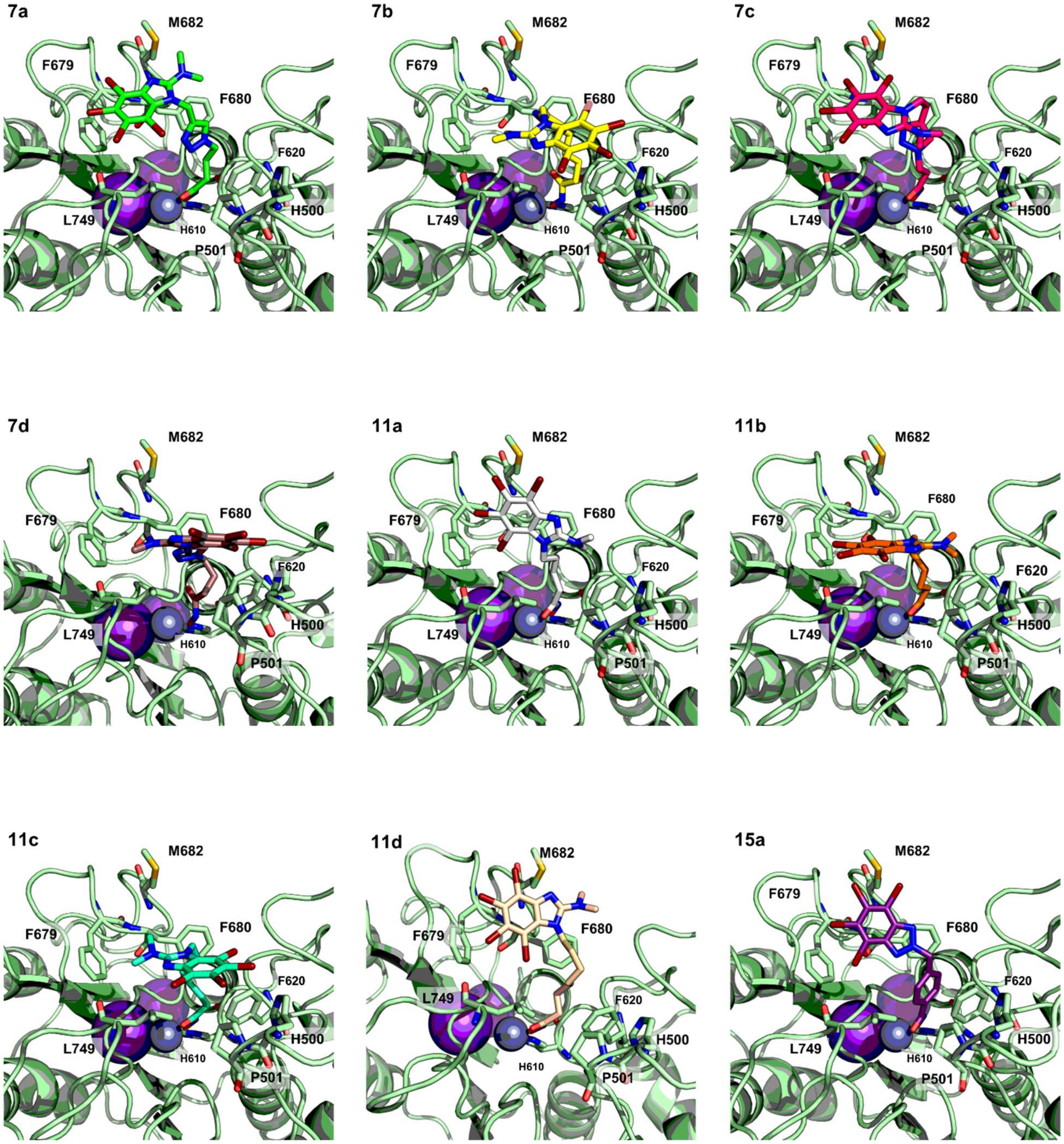
7a
7b
7c
7d
11a
11b
11c
11d
15a
M682
F679
F680
F620
H500
L749
H610
P501

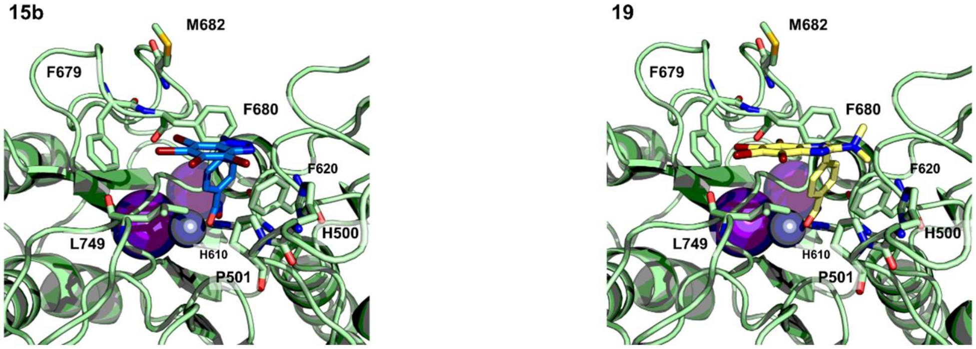

Figure 7S: PyMOL stick and cartoon representation of the best docking poses obtained for the set of compounds in HDAC6. For the sake of clarity only heavy atoms are shown.

Table 1S: RMSD (Å) values of the set of compounds in complex with CK2.

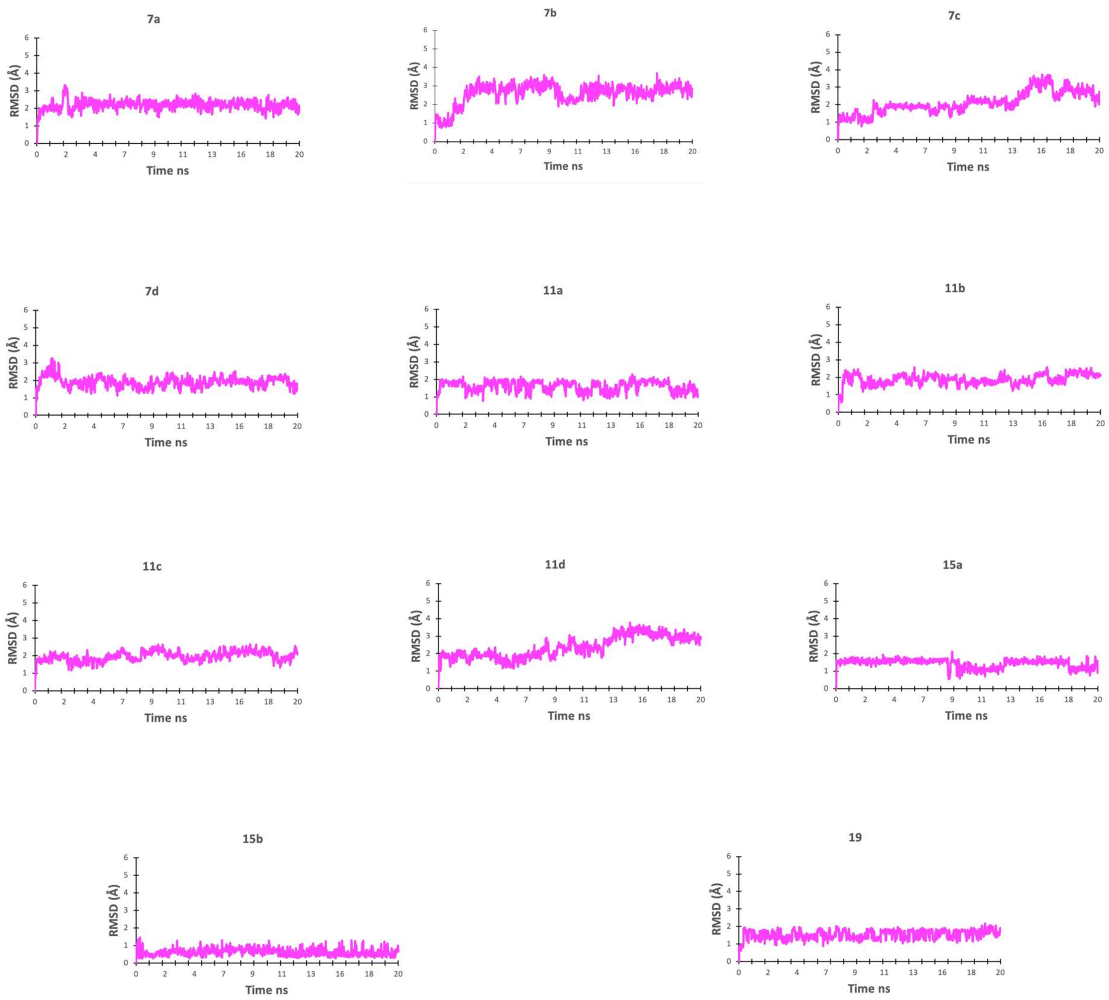

Table 2S: RMSD (Å) values of the set of compounds in complex with HDAC1.

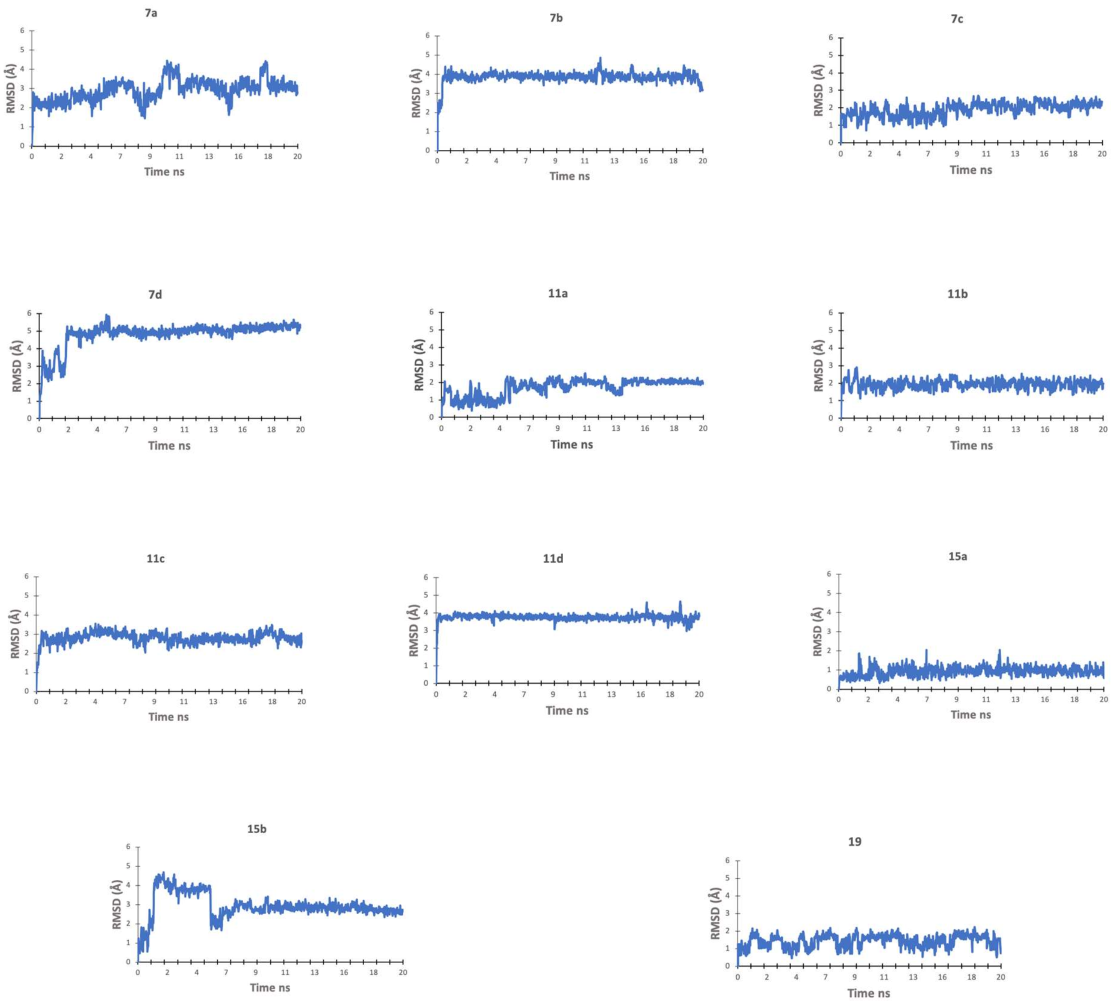

Table 3S: RMSD (Å) values of the set of compounds in complex with HDAC6.

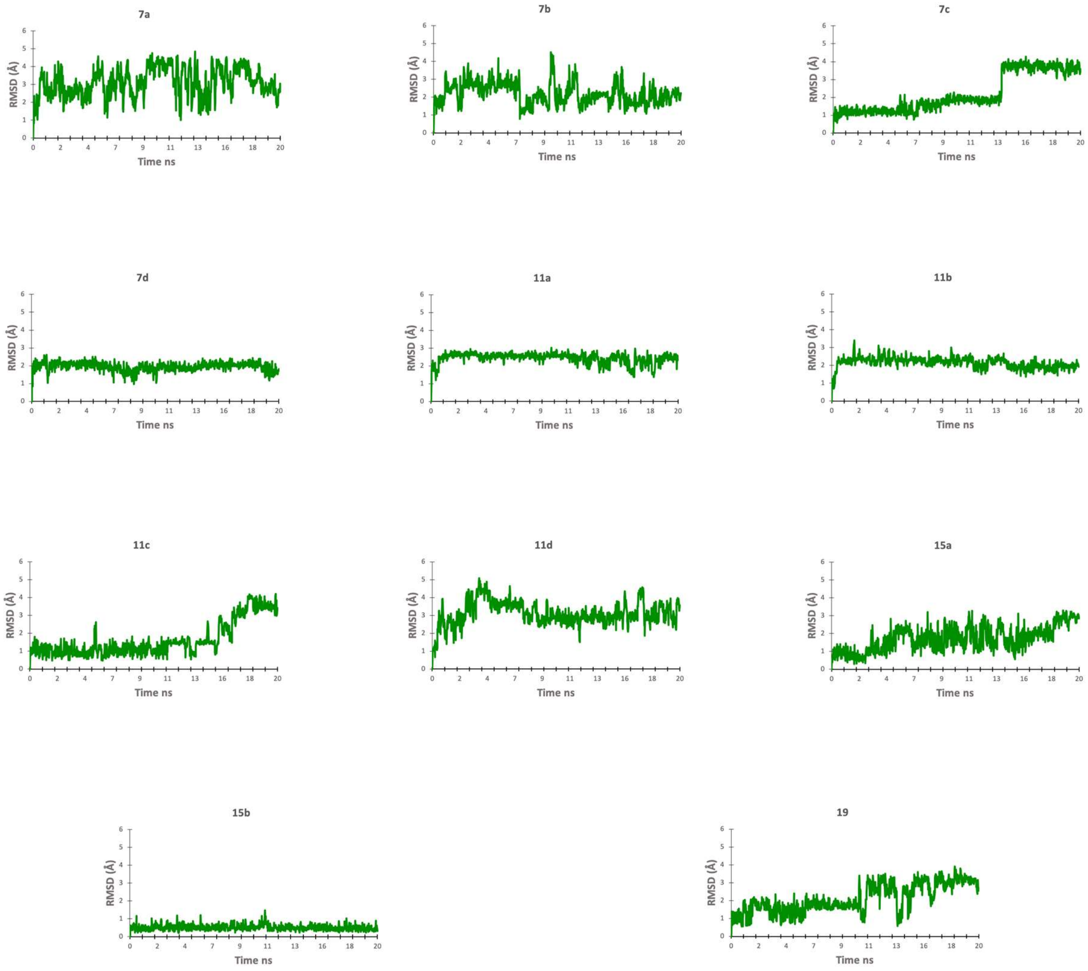

Table 4S. LC_{50} values for **7c**, **11a**-**d** and **19** towards human tumor and pseudonormal cell lines in vitro

Compound	LC_{50} values of compounds for cell line, μM (M±SD)						
	Jurkat	MCF-7	HCT-116	HEK293	HL-60	HL-60/adr	HL-60/vinc
**7c**	10,69±1,50	15,66±2,54	4,22±0,46	23,33±4,44	7,67±1,18	16,23±1,70	17,56±1,25
**11b**	10,04±1,15	9,97±0,99	1,90±0,24	13,61±0,76	6,59±0,96	10,63±1,55	12,67±3,50
**11c**	5,63±0,78	9,02±0,90	3,10±0,37	8,41±1,28	4,69±0,54	8,40±1,23	2,49±1,11
**11d**	14,17±1,83	27,75±6,06	16,87±3,90	>30	29,69±4,40	15,10±1,59	>30
**15a**	>30	13,66±2,67	8,67±1,02	>30	>30	>30	>30
**19**	21,80±3,17	13,55±0,78	11,30±1,01	24,47±8,335	>30	>30	26,58±6,05

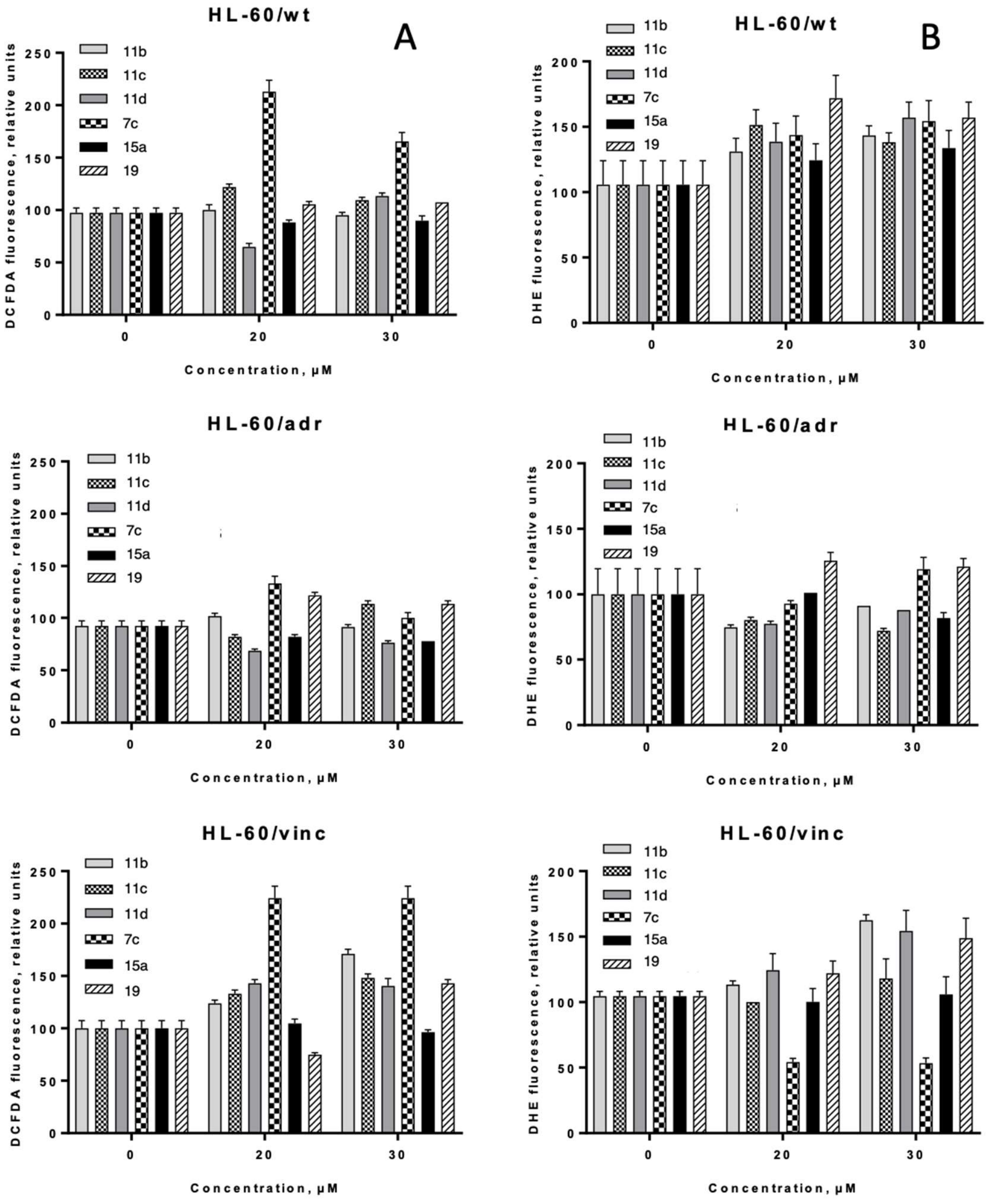

Figure 8S. Induction of reactive oxygen species (ROS) by the indicated CK2-HDAC dual inhibitors (10 µM and 30 µM, 24 hours) in HL-60, HL-60/adr and HL-60/vinc leukemia cells at 24h incubation DCFDA (A) and DHE (B) assays were used to visualize hydrogen peroxide and superoxide production and the respective fluorescence was measured at the single cell level in FACS analyses. One of three experiments delivering comparable data is shown.
